# Atherosclerosis: from lipid-lowering and anti-inflammatory therapies to targeting arterial retention of ApoB-containing lipoproteins

**DOI:** 10.3389/fimmu.2025.1485801

**Published:** 2025-06-09

**Authors:** Gala Araujo, Leidy Marian Valencia, Agata Martin-Ozimek, Yosdel Soto, Spencer D. Proctor

**Affiliations:** ^1^ Metabolic and Cardiovascular Diseases Laboratory, Division of Human Nutrition, Alberta Diabetes Institute, University of Alberta, Edmonton, AB, Canada; ^2^ Department of Immunobiology, Centre for Molecular Immunology, Havana, Cuba

**Keywords:** cardiovascular disease, atherosclerosis, innate immunity, adaptive immunity, proteoglycans, immunotherapies, monoclonal antibodies, extracellular matrix

## Abstract

Atherosclerosis is a chronic inflammatory disease characterized by the accumulation of lipids, fibrous elements, and cellular debris in the blood vessels. The response-to-retention hypothesis, the leading theory on the pathogenesis of this cardiovascular disease, describes the initial event in atherosclerosis as when Apolipoprotein B-containing lipoproteins, including endogenous and dietary-derived lipoproteins, bind to the inner arterial wall, the tunica intima. The subsequent lipoprotein modifications trigger an immune response that promotes atherosclerotic plaque formation. Despite the prevalence of atherosclerosis globally, and its vascular nature, therapies directed to the artery wall are limited. Immunotherapies, most notably monoclonal antibodies (mAbs), are of special interest due to their high specificity, reliability and proven success in a variety of diseases. However, current mAbs for atherosclerosis tend to target disease risk factors, notably inflammation and circulating lipoprotein levels, rather than address the root cause of atherosclerosis. These treatments result in a phenomenon known as residual risk, defined by the occurrence of severe cardiovascular events, including myocardial infarction, during treatment. Per the “response to retention” hypothesis, a plausible strategy for atherosclerosis would be blocking cholesterol retention *per se* at the arterial extracellular matrix level to complement lipid-lowering therapies. One such immunotherapy is the chP3R99 mAb, which can bind to pro-atherogenic proteoglycan sugar branches, thus competitively inhibiting lipid retention at these sites. The aim of this review is twofold: 1) To provide a summary of mAbs and other therapies used for atherosclerosis treatment, focusing on anti-inflammatory and lipid-lowering therapies, and 2) To review data on the structural characteristics, theory, and therapeutic effect of the chP3R99 mAb.

## 1 Introduction

Atherosclerosis involves the gradual accumulation of cholesterol and the development of fat-rich fibrous plaques within the tunica intima, the innermost layer of the arterial wall. A crucial feature of atherosclerosis is the presence of low-grade chronic inflammation, which occurs as a protective response to proatherogenic lipoproteins infiltrating the arterial wall (1–3). The response-to-retention hypothesis of atherosclerosis states that atherogenesis is triggered by the subendothelial retention of Apolipoprotein B (ApoB)-containing lipoproteins such as low-density lipoprotein (LDL), lipoprotein (a) (Lp(a)), and, triglyceride-rich lipoproteins, including the dietary-derived chylomicron remnants (1, 4, 5). As the plaque develops, it calcifies, and the fibrous cap is degraded, significantly increasing the risk of rupture or thrombosis. This, in turn, can cause ischemia, myocardial infarction, and death (6). Despite the prevalence of atherosclerosis, there are relatively few effective therapies, most of which are focused on modulating risk factors rather than targeting the artery wall. Immunotherapies are becoming increasingly popular research avenues for atherosclerosis. Monoclonal antibodies (mAbs) are of specific interest due to their high specificity and sensitivity, relatively low side effects, and, history of therapeutic success (7). Currently, commercially available mAbs for atherosclerosis fall into the category of anti-inflammatory or lipid-lowering therapies. Anti-inflammatory mAbs decrease inflammation, thus reducing the burden on the blood vessels and limiting plaque formation. The CANTOS study was a landmark clinical trial that used canakinumab to reduce the levels of interleukin-1β (IL-1β), a critical pro-inflammatory cytokine in atherosclerosis (8–11). However, while inflammation and cardiovascular disease (CVD) incidence were reduced in the treatment group compared to the placebo, there were no significant changes in participant mortality. This phenomenon of life-threatening CVD events during treatment is known as residual risk, which indicates the persistent need for complementary treatments for atherosclerosis (8). Lipid-lowering mAbs function by targeting cholesterol synthesis mediators and LDL directly. However, similar to anti-inflammatory agents, they have encountered challenges in reducing all cardiovascular event incidences across patients (12). Research on mAbs targeting extracellular matrix (ECM) components has attracted significant attention due to the role of the ECM in the early stages of atherogenesis (13, 14). One such example is the chP3R99 mAb, which can bind to proteoglycan side chains and interfere with lipoprotein binding. Through competitive inhibition, the chP3R99 mAb inhibits lipoprotein retention and the subsequent formation of an atherosclerotic plaque, thus acting as a potential new therapy for atherosclerosis (15). Despite strong preclinical evidence supporting the efficacy of chP3R99 mAb in atherosclerosis management, information integrating its structural characteristics, functional basis, challenges, and future prospects are limited in existing literature. Here, we raise essential context and discuss the theoretical underpinnings of the chP3R99 mAb and its potential as an immunotherapy.

## 2 Pathogenesis and evolution of atherosclerosis

Atherosclerosis is the predominant form of CVD globally (16). Atherogenesis begins in childhood, as lipids and fibrous elements accumulate in medium and large-caliber arteries (17). Atherosclerosis progresses silently for decades until causing clinical events that can be fatal (18, 19). Globally, imaging-based studies estimate that approximately 50% of individuals over the age of 40 exhibit subclinical carotid atherosclerosis, with prevalence rates rising steadily (20). Economic development, rapid urbanization and globalization have promoted atherosclerosis by facilitating dangerous lifestyle choices, such as diets rich in saturated fat or reduced physical activity (21). Several non-modifiable risk factors are linked to atherosclerosis development, such as age, family history, and sex (22). Conversely, modifiable risk factors include hypercholesterolemia, obesity, hypertension, smoking, diabetes, and certain pathogen-related infections such as chlamydia (23). Of these factors, hypercholesterolemia plays a dominant role in the onset and progression of atherosclerosis, the risk of which increases with proatherogenic lipoprotein levels (24, 25).

One of the historical hypotheses describing the pathogenesis of atherosclerosis is the response-to-injury hypothesis (26). According to this theory, atherosclerosis results from endothelial damage caused by higher shear stress at arterial bends and bifurcations, leading to higher permeability to lipoproteins. While endothelial damage and lipoprotein levels are proven to be considerable risk factors for atherosclerosis progression, inconsistent evidence supports this theory. Notably, the lack of atherosclerotic remodeling in areas of endothelial damage and the presence of remodeling in areas void of endothelial damage challenges the response-to-injury hypothesis. Due to these observations, the response-to-retention theory has been established as the most probable mechanism to describe the pathogenesis of atherosclerosis, providing a more active role for the ECM in atherosclerosis onset (1, 27). Thus, the key initiating event of atherosclerosis is the subendothelial retention of LDL and other ApoB-containing lipoproteins like Lp(a) and remnant lipoproteins (1, 28).

ApoB-containing lipoproteins primarily traverse the arterial endothelium via transcytosis, a process governed by particle size and receptor interactions. Seminal studies by Simionescu and colleagues established that particles ≤70 nm in diameter—including LDL, Lp(a), and smaller triglyceride-rich lipoproteins—cross the endothelial barrier into the intima, while larger particles like very-low-density lipoproteins (VLDL) and chylomicrons are excluded due to size constraints (29, 30). Under physiological conditions, LDL (18-25nm) transcytosis occurs through LDL receptor (LDLR)-dependent pathways and caveolae-mediated transport. More recently it was demonstrated that the latter mechanism is facilitated by the activin receptor-like kinase 1 and the scavenger receptor B1 (31–34). Similarly, triglyceride-rich lipoprotein remnants [such as VLDL remnants (35–50 nm), chylomicron remnants (30–80 nm), and intermediate-density lipoproteins (25–35 nm)], can access the intima via scavenger receptor-mediated active transcytosis and updated to include particles of size ≤80nm (35–38). Notably, while Lp(a) (25–70 nm) particles fall within this size range and share structural similarities with LDL, these particles exhibit a weaker binding to LDLR. Therefore, Lp(a) trans-endothelial transport mechanisms remain poorly understood and their interaction with plasminogen receptors and scavenger receptors may play a more significant role in this process (39).

In pathological states, like sustained hypercholesterolemia and inflammation, the endothelial permeability is increased and transcytosis of ApoB-containing lipoproteins other than LDL is enhanced (40). A novel mechanism for triglyceride-rich lipoproteins arterial delivery mediated by the induction of lipid droplet formation in the endothelium has been described recently (41–43). Thus, while LDL dominates intimal delivery, smaller remnants, and particularly the infiltration of chylomicron remnants in metabolic disorders and the postprandial state, further contribute to arterial lipid accumulation (35, 38, 44–46). Although these mechanisms occur without prior endothelial damage, permissive conditions like endothelial dysfunction, inflammation, and structural alterations, such as the absence of a confluent luminal elastin sheet, and exposure of arterial proteoglycans, not only increase ApoB-containing lipoproteins delivery in the intima but also accelerate the subendothelial deposition of lipids and contribute to the onset and progression of atherosclerosis (40, 45, 47, 48).

Lipoprotein retention in the arterial intima is a hallmark of early atherogenesis, driven by electrostatic interactions between glycosaminoglycan (GAGs) chains on proteoglycans and basic residues (arginine/lysine) within ApoB (49–51). This molecule exists as two isoforms: ApoB100 (4536 amino acids) and ApoB48 (N-terminal 2152 amino acids), both of which contribute to atherogenicity despite structural differences (44, 52–55). Although the carboxyl-terminal Site B (residues 3359–3369) of ApoB100 is the primary proteoglycan-binding domain, ApoB48 compensates for the absence of this region via an alternative binding site (Site B-Ib) located at the amino-terminal region (56, 57). In ApoB100, Site B-Ib is masked by the carboxyl terminus, whereas truncation in ApoB48 exposes this region, facilitating proteoglycan binding. This mechanism supports the response-to-retention hypothesis for different classes of lipoproteins, explaining why both isoforms are (at least) equally atherogenic and contribute to lipid accumulation and vascular disease progression (40, 44, 57, 58).

**Figure 1:**
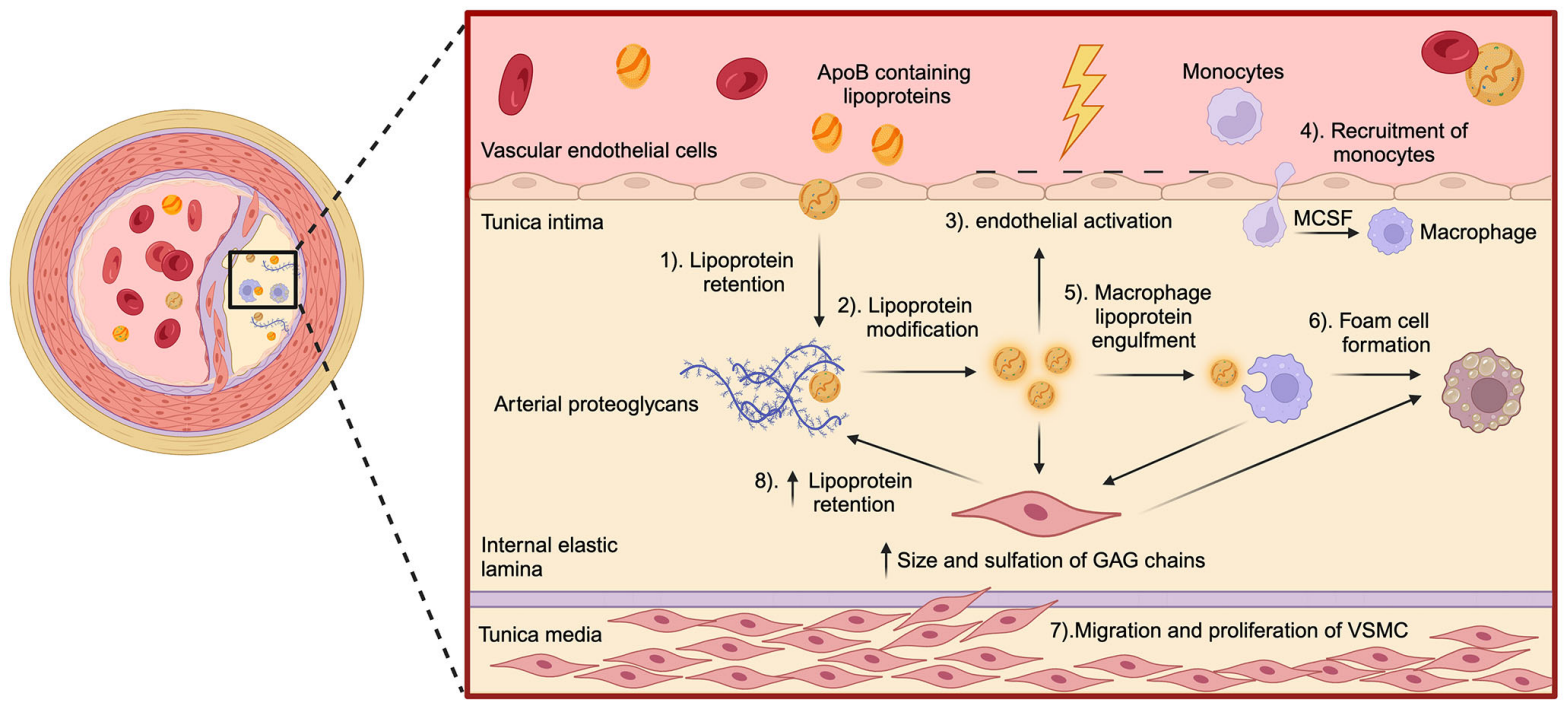
Key steps in the pathogenesis of atherosclerosis. The schematic illustrates the major molecular and cellular events involved in the development of atherosclerosis within the artery wall. (1) As per the response to the retention hypothesis, the process begins with the retention of ApoB-containing lipoproteins in the tunica intima, facilitated by interactions with arterial proteoglycans. (2) Once trapped, these lipoproteins undergo structural modifications, such as oxidation and aggregation. (3) Subsequently, these modifications trigger endothelial activation, leading to (4) the recruitment of monocytes into the intima. Monocyte recruitment is mediated by factors like macrophage-colony stimulating factor, released by endothelial cells. Within the intima, monocytes differentiate into macrophages, (5) which then engulf modified lipoproteins, (6) transforming into lipid-laden foam cells. The effector mechanisms of macrophages contribute to oxidative stress within the vasculature, which in turn (7) promotes the migration of vascular smooth muscle cells (VSMC) from the tunica media to the intima. In the intima, VSMC proliferate and contribute to plaque development by contributing to foam cell formation. (8) Increased size and sulfation of glycosaminoglycans chains within arterial proteoglycans produced by VSMC further enhance ApoB-containing lipoprotein retention accelerating plaque progression.

Once retained in the arterial wall, lipoproteins undergo different modifications, including oxidation, enzymatic modifications, and aggregation (1). Oxidized lipoproteins release bioactive molecules, such as oxidized phospholipids, which directly activate endothelial cells (2, 59). Arterial tissue-resident macrophages, derived from embryonic CX3CR1+ precursors, are crucial for detecting modified lipids and maintaining vascular homeostasis. This population is established in the vascular wall during mid-gestational development and possess self-renewing capacity through local proliferation (60). They initiate an inflammatory response when exposed to persistent stimuli, such as oxidized lipids (61, 62) (**Figure 1**). Macrophages internalize modified lipoproteins via scavenger receptors (e.g., CD36, scavenger receptor-A), leading to intracellular cholesterol accumulation and their transformation into foam cells, a hallmark of atherosclerosis across all stages of the pathology (63). Due to their limited self-renewing capacity, tissue-resident macrophages cannot sustain plaque expansion during disease progression (64). Consequently, activated endothelial cells upregulate adhesion molecules and chemotactic factors, recruiting monocytes and lymphocytes from the bloodstream into the arterial intima (65). Within the artery wall, infiltrating monocytes differentiate into macrophages under the influence of growth factors secreted by endothelial cells and resident macrophages. This differentiation amplifies the expression of pattern recognition receptors, particularly scavenger receptors and toll-like receptors (TLRs), further perpetuating lipid uptake and inflammatory signaling (66, 67).

Vascular smooth muscle cells (VSMCs) are also key players in atherosclerosis. Several growth factors and cytokines produced by macrophages, mainly the platelet-derived growth factor, contribute to the migration of VSMCs and subsequent differentiation in the tunica intima. Hence, VSMCs acquire a synthetic phenotype with increased production of collagen, elastic fibers and fibrous tissue (68). Proliferating VSMCs, along with the production of ECM, generate a fibrous layer that covers the developing atherosclerotic plaque, surrounding the lesion and preventing its rupture (69). However, these cells also produce pro-atherogenic proteoglycans, almost exclusively made up of chondroitin sulfate (CS), characterized by elongated GAGs chains, changes in their sulfation pattern, and increased content of sulfate groups, altogether increasing their affinity and retention to lipoproteins (70, 71). VSMCs also acquire the ability to internalize modified lipoproteins through the expression of several scavenger receptors, accounting for the majority of foam cells in the atheroma (72, 73). Furthermore, VSMCs of the intima express major histocompatibility complex II (MHC-II) molecules and, therefore, can also behave as antigen-presenting cells (APCs) (74). The final development of the atherosclerotic lesion involves the production of several degradative enzymes, which make the fibrous layer prone to rupture due to the destruction of the ECM and lead to the formation of a life-threatening thrombus (19, 75).

### 2.1 Innate and adaptive immunity

#### 2.1.1 Innate immunity as a key player in atherogenesis

Subendothelial lipid accumulation and the subsequent oxidative and enzymatic modifications further stimulate tissue-resident macrophages and endothelial cells to generate inflammatory mediators like cytokines, chemokines, growth factors, and reactive oxygen/nitrogen species, contributing to the initial steps of atherogenesis (76). These changes increase endothelial damage along with the expression of adhesion molecules (77). As mentioned, the activation of the endothelium leads to the extravasation of monocytes to the intima, which then differentiate into macrophages activated by the monocyte colony-stimulating factor. Macrophages exhibit robust phagocytic activity, secrete a wide range of soluble factors, and are involved in ECM remodeling, actions which are central to their role in atherosclerosis progression. Large numbers of macrophages are found in atherosclerotic plaques, especially at the shoulders of lesions, expressing an inflammatory M1 phenotype (78). Activated macrophages phagocytose modified lipoproteins, as well as large and aggregated particles, in an unregulated manner, leading to the accumulation of cholesterol in its cytoplasm to form the foam cells that characterize this pathology (61, 79).

Additionally, macrophages can internalize modified LDL through receptor-mediated phagocytosis and pinocytosis, among other mechanisms, both actin-dependent and independent (80). Macrophages can also express TLRs that can recognize and internalize oxidized LDL (oxLDL) and, in turn, trigger signaling cascades that activate macrophages themselves (81). Oxidized lipoproteins act as damage-associated molecular patterns, stimulating TLRs in macrophages, which aggravates inflammation in the plaque (82). Macrophages recognize and internalize oxLDL via an array of scavenger receptors, which, unlike LDLR, are not inhibited by high intracellular cholesterol concentrations (83). Cholesterol crystals inside macrophages are the trigger for the assembly and activation of the NOD-like receptor P3 (NLRP3) inflammasome, responsible for activating proinflammatory cytokines such as IL-1β and IL-18 (84). Macrophages account for an essential source of vasoactive molecules, endothelin and various eicosanoids that promote the recruitment of leukocytes to the arterial wall and contribute to inflammation (85). The main soluble factors produced by macrophages include macrophage colony-stimulating factor, platelet-derived growth factor, transforming growth factor-β (TGF-β), tumour necrosis factor-α (TNF-α), and interleukins (IL), IL-1β, IL-6, and IL-8 (86). Likewise, in the presence of interferon-gamma (IFN-γ), macrophages produce other mediators such as monocyte chemoattractant protein-1, IL-12, and IL-18. Together, these molecules recruit and activate more leukocytes, contributing to local inflammation and apoptosis that characterize advanced lesions’ lipid core. Lastly, macrophages secrete ECM-degrading enzymes such as matrix metalloproteinases, lysosomal proteases including cathepsins F and S, collagenases, heparinases, and sulfatases. The production of these enzymes further contributes to the pathophysiology of atherosclerosis by releasing cytokines and growth factors inactively sequestered in the extracellular space (87).

#### 2.1.2 Contribution of adaptive immunity to the development of atherosclerosis

**Figure 2:**
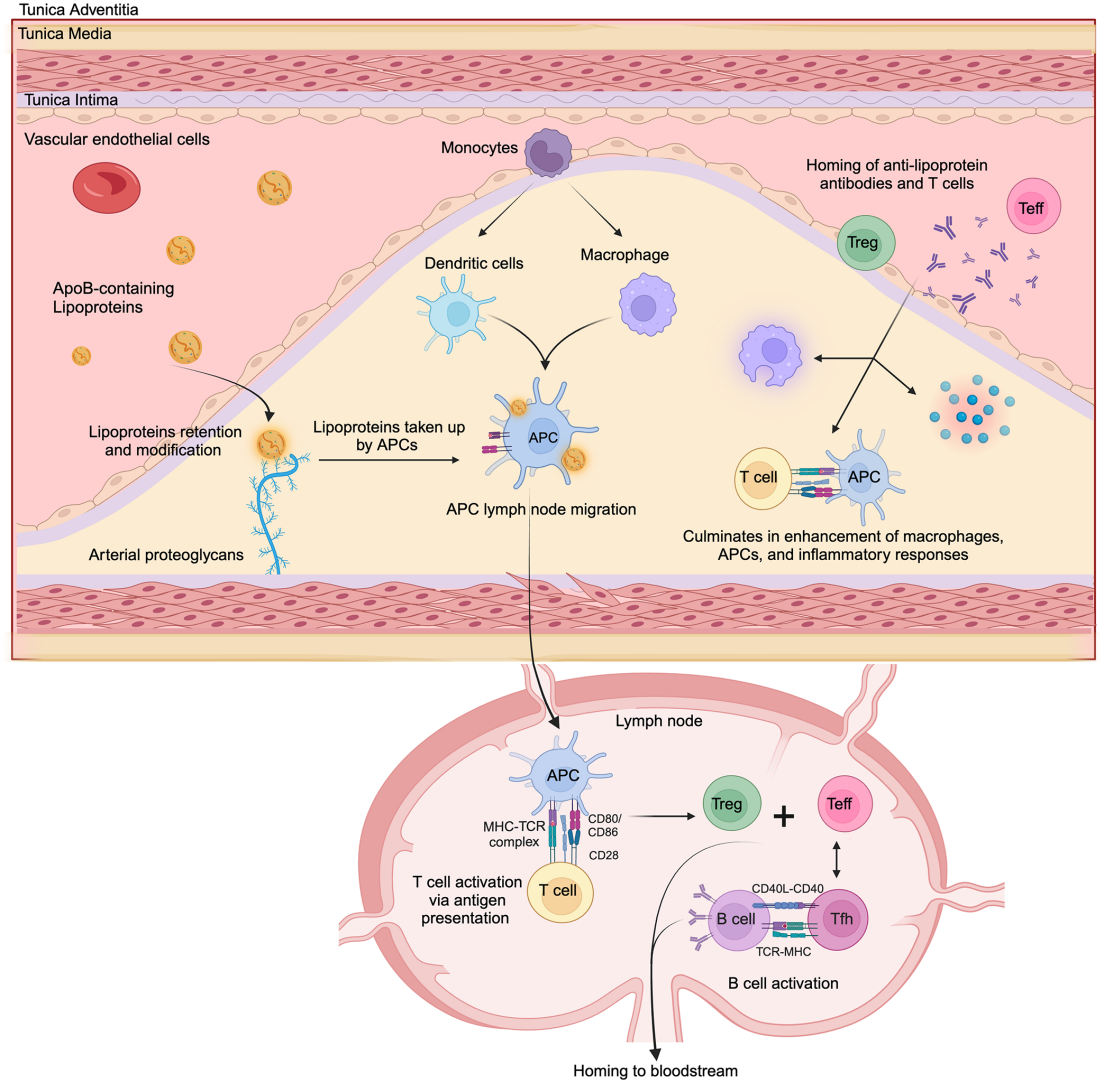
Simplified model of key immune events in atherosclerosis development. ApoB-containing lipoproteins retained in the intimal layer of the artery wall are modified and subsequently taken up by antigen-presenting cells (APCs), including dendritic cells and macrophages. These APCs then migrate to lymphoid organs, including draining lymph nodes. Within the lymph node, APCs present processed lipoprotein-derived peptides to T cells via MHC-TCR interaction, along with costimulatory signals (CD80/CD86 - CD28), leading to T cell activation and differentiation into effector T cells (Teff) and regulatory T cells (Treg). Following activation, T cells can differentiate into CD4+ T helper subsets, including pro-inflammatory phenotypes like Th1 and Th17 cells, or cytotoxic CD8+ T lymphocytes that contribute to the pathogenesis of atherosclerosis. Also, CD4+ follicular helper T cells interact with B cells (via CD40L-CD40 and TCR-MHC), promoting their activation and antibody production. Anti-lipoprotein antibodies, effector T cells, regulatory T cells and acute-phase inflammatory proteins are recruited to the plaque. Within the plaque, interactions between activated T cells that have homed back to the lesion with APCs create a pro-inflammatory feedback loop, amplifying local inflammation and lesion progression.

The role of adaptive immunity in atherosclerosis has been extensively studied in animal models and humans, with a particular emphasis on immune responses directed to oxidation-specific epitopes derived from oxLDL (88). The transition to adaptive immunity in the vasculature is initiated when retained or modified lipoproteins are internalized by professional APCs, such as dendritic cells (DCs) and macrophages. Following antigen uptake, DCs migrate to secondary lymphoid organs—including draining lymph nodes and the spleen—where they prime naïve T cells (**Figure 2**) (89). Vascular antigens are processed by these APCs and the resulting peptides are presented in the context of MHC-I and MHC-II molecules to CD8+ and CD4+ T cell, respectively (90–93). Under homeostatic conditions, DCs in healthy arteries may present self-antigens in the absence of co-stimulatory signals, promoting T cell tolerance or anergy (94). This aligns with studies showing that T cells activated by non-professional APCs fail to upregulate co-stimulatory molecules like CD80/CD86, leading to functional unresponsiveness upon re-stimulation (94). In atherosclerotic plaques, however, DCs undergo maturation triggered by pro-inflammatory mediators such as TLR agonists, danger-associated molecular patterns, and cytokines (e.g., IFN-γ, TNF-α). Mature DCs upregulate MHC-II, co-stimulatory molecules (CD80, CD86, CD83), and chemokine receptors (e.g., CCR7) (95–97). In advanced lesions, the number of DCs is increased compared to early lesions where they accumulate in rupture-prone regions, forming clusters with T cells (98).

While the precise antigen epitopes driving adaptive T-cell responses in atherosclerosis remain under investigation, growing evidence highlights native and oxidized ApoB-derived epitopes as critical contributors (99–101). Oxidation-specific epitopes, such as malondialdehyde and 4-hydroxynonenal-adducted lysine residues on ApoB, have been proposed as key players in T-cell activation across experimental and human studies (88, 91, 102–104). However, recent advances based on peptide-specific tetramer staining and single-cell transcriptomics have identified CD4+ T cells reactive to native ApoB in murine and human atherosclerosis, challenging the exclusivity of modified ApoB in this process (105, 106). Notably, T-cell epitopes shared by ApoB100 and ApoB48 have been identified (107, 108) demonstrating that T-cell activation in atherosclerosis is not restricted to ApoB100-derived antigens (109).

In atherosclerotic plaques, most CD4+ T lymphocytes express αβ T-cell receptors and exhibit an effector memory phenotype, although subpopulations expressing γδ T-cell receptors are also present in smaller numbers (110, 111). Upon antigenic stimulation, CD4+ T lymphocytes differentiate into various T-helper (Th) subsets, each with distinct functional characteristics. These subsets include pro-inflammatory phenotypes such as Th1, Th2, and Th17 cells, as well as follicular helper T (Tfh) cells and regulatory T cells (Tregs) (112). The different Th subsets influence the progression of atherosclerosis through various mechanisms. Notably, Tfh cells promote immune activation by supporting B-cell maturation and high-affinity IgG antibody production within germinal centers (100). In the plaque, a predominant Th1 phenotype is observed, characterized by the secretion of IFN-γ and TNF-α, which drives plaque progression and instability, as demonstrated in mouse models and human studies (113–116). In contrast, Tregs suppress inflammation via IL-10 and TGF-β, though their frequency declines as lesions advance (117, 118). The roles of Th2 (IL-4/IL-5) and Th17 (IL-17) cells remain controversial, with evidence supporting both pro- and anti-atherogenic effects (119, 120). Additionally, natural killer T cells, activated by lipid antigens presented via CD1 molecules, further amplify pro-inflammatory cascades (119, 121).

On the other hand, studies in ApoE^−/−^ mice that depleted CD8+ cytotoxic T lymphocytes (CTLs) found reduced lesion area, lipid content, macrophage infiltration, and necrotic core size (122). This suggests that CTLs also contribute to atherosclerosis progression by promoting necrotic core formation through the induction of apoptosis in macrophages, VSMCs, and endothelial cells (123, 124). Mechanistically, lesional CTLs express perforin and granzyme B, which colocalize with apoptotic vascular cells. Genetic deletion of these cytotoxic molecules further confirmed their role in necrotic core expansion (122). Interestingly, these molecules may also attenuate atherogenesis by suppressing APCs and other effector T cells, a regulatory effect that appears more prominent in the early stages of the disease (124). Beyond direct cytotoxicity, CD8+ T cells exacerbate plaque inflammation by secreting TNF-α (125) and amplifying systemic monocytosis via IFN-γ-mediated bone marrow activation (122). While some lesional CD8+ T cells recognize ApoB-derived peptides, the specific antigen driving their activation remains elusive, as is the case for CD4+ T cells (123, 126). Collectively, these findings underscore CD8+ T cells as central mediators of plaque vulnerability, linking adaptive immune responses to impaired efferocytosis, sustained inflammation, and necrotic core progression (122).

B cells are scarce within atherosclerotic plaques but accumulate in periadventitial lymphoid infiltrates near advanced lesions, indicating localized adaptive immune responses (127, 128). B cell subsets exhibit opposing effects on atherosclerosis progression. B1 cells secrete natural IgM antibodies targeting oxidation-specific epitopes, such as phosphocholine on oxLDL, which reduce inflammation and inhibit foam cell formation (91, 129, 130). Notably, approximately 30% of natural IgM antibodies are directed against those epitopes, which are shared by apoptotic cells, bacterial pathogens, and oxidized lipoproteins (131). Conversely, B2 cells have been shown to promote atherosclerosis through proinflammatory IgG production and T cell activation (122, 132, 133). While there is a consensus that anti-oxLDL IgM antibodies are atheroprotective, the role of IgG subclasses remains ambiguous, with studies implicating both pathogenic and protective effects (129, 134–137). Recent work emphasizes the critical role of T-cell–B-cell interactions in modulating the nature of humoral responses in atherosclerosis. CD4+ Tfh cells play a pivotal role by facilitating B cell maturation and antibody class-switching. Depending on the context, these interactions within germinal centers or tertiary lymphoid structures can drive the production of either pro-atherogenic or atheroprotective antibodies, highlighting their dual role in plaque formation (100).

Following activation in secondary lymphoid organs, T cells enter systemic circulation and home to atherosclerotic plaques through mechanisms described for monocyte extravasation (112, 138). In this case, the process involves interactions of adhesion molecules from the inflamed endothelium like selectins (E- and P-selectins) and integrins (e. g. Vascular Cell Adhesion Molecule-1) with their counterparts expressed in activated T cells (e.g. Very Late Antigen-4) (138). After transmigrating into the lesion, T cells are reactivated by local APCs, which in turn trigger cytokine secretion by CD4+ Th cells, enhance pro-inflammatory macrophage activity, and promote cytotoxic activity by CTLs (139).

It is proposed that antibodies (~12nm) can be recruited to the sub-endothelium by similar pathways that are active for lipoprotein permeability (<80nm). We have shown recently that antibodies can be detected in the vasculature within minutes of infusion using different model species (140–142). As plaques advance, sustained inflammatory signaling disrupts endothelial integrity by opening intercellular junctions and creating transient gaps leading to increased vascular permeability (143, 144). This enables the leakage of large molecules into the atheroma, including antibodies, acute-phase proteins (e.g. C-reactive protein), complement components, and larger lipoprotein particles (145, 146). The influx of these mediators, combined with the ongoing recruitment of immune cells, creates a self-perpetuating cycle of lipid accumulation, inflammation, and plaque destabilization (147).

In summary, adaptive immunity in atherosclerosis is a complex process. T cells, particularly CD4+ and CD8+ subsets, play varied roles, from promoting inflammation to inducing cytotoxicity. B cells and antibodies also exert both pro- and anti-atherogenic effects. A deeper understanding of these mechanisms is crucial for developing targeted therapeutic strategies.

#### 2.1.3 Immune checkpoints as mediating factors in atherosclerosis

Co-stimulatory molecules and immune checkpoint proteins have been reported to be pivotal in modulating atherogenesis (148). Immune checkpoints found on APCs and T cells regulate the immune response and prevent overstimulation of the immune system. The role of immune checkpoints in managing an immune response’s regulation, inhibition, severity, and length has been well documented. As mentioned, T cells become activated through interactions with APCs (149). However, a second signal is needed for T cell activation in addition to antigen presentation. This second signal can occur through the co-stimulation of receptors on T cells and stimulatory molecules on APCs (149), such as the co-stimulation of the CD28 receptor on T cells binding to CD80/86 on APCs. This signal is necessary for the downstream activation of signaling pathways, specifically the PI3K/Akt pathway (149, 150). Activating the PI3K/Akt pathway further stimulates T cells’ differentiation, proliferation and survival, which can have critical effects during atherosclerosis.

Moreover, cytotoxic T-lymphocyte antigen (CTLA)-4 inhibits this co-stimulation, providing another avenue for immune regulation (150). Conversely, the interaction between programmed cell death protein 1 (PD-1) on T cells and the programmed death ligands 1 and 2 (PD-L1, PD-L2) on blood cells and phagocytes, respectively, acts as a significant immune checkpoint that reduces T cell activity (149). This process occurs through dephosphorylation and subsequent inhibition of the PI3K-Akt pathway (149).

## 3 Therapeutic strategies targeting atherosclerosis

### 3.1 Anti-inflammatory therapies and monoclonal antibodies

The CANTOS trial was a large-scale clinical trial that described the use of canakinumab, a mAb inhibitor of the pro-inflammatory cytokine IL-1β. Canakinumab directly binds to IL-1β, thereby preventing Il-1β mediated inflammation and reduced the risk of recurrent cardiovascular events (151). The CANTOS trial used a comprehensive randomized, blinded, placebo-controlled study design to follow 10,061 patients across 39 nations with previous reports of myocardial infarction and increased high-sensitivity C-reactive protein (hsCRP) levels from 2011 to 2017 (151, 152). The study found that over 3.7 years, the placebo group experienced 4.50 events per 100 person-years, while the 300 mg canakinumab treatment group experienced 3.90 events per 100 person-years (151). Thus, 300 mg canakinumab administration reduced recurrent major adverse cardiovascular events (MACE) by 0.60 per 100 person-years compared to the placebo cohort (151). Interestingly, however, the canakinumab treatment group reported higher neutropenia cases than the placebo group. Similarly, the canakinumab group also exhibited higher rates of infection or sepsis-related deaths relative to the placebo group. Specifically, the canakinumab group experienced an incidence rate of 0.31 of sepsis and infection-related deaths per 100 person-years versus 0.18 events per 100 person-years for the placebo group (151). Additionally, thrombocytopenia was more frequent among those receiving canakinumab than the placebo group, but there were no notable differences in the incidence of hemorrhages (151). As such, commercial approval for canakinumab and other notable IL-1β therapies, such as gevokizumab and mavrilimumab, has yet to be approved. Similarly, preliminary studies using infliximab and certolizumab anti-TNF-α therapies found reduced monocyte and neutrophil activity and improved endothelial and arterial wall function (153). Moreover, various pro-inflammatory cytokine therapies target the NLRP3 inflammasome, indirectly inhibiting IL-1β and TNF-α activity. A notable example is the synthetic NLRP3 inhibitor, MCC950, described by Coll et al. in 2015 (154). MCC950 works by binding to the NLRP3 protein, thereby preventing inflammasome assembly (154). This interaction suppresses IL-1β activation by inhibiting caspase-1 and caspase-11 pathways (155). A 2021 study by Zeng et al. described the use of MCC950 in atherosclerosis using ApoE**^-/-^** mice (156). Following MCC950 administration, the group found evidence of decreases in atherosclerotic plaque size, macrophage levels, and pro-inflammatory cytokines, specifically IL-1β and IL-18 (153). Another prominent NLR3P inflammasome inhibitor, the ketone β-hydroxybutyrate, prevents potassium (K+) efflux, thereby inhibiting apoptosis-associated speck-like protein with a caspase recruitment domain oligomerization, which is necessary for caspase-1 activation. A 2015 investigation reported that β-hydroxybutyrate use in mice with NLRP3-related disorders significantly reduced pro-inflammatory cytokines, specifically ILs (157).

However, anti-inflammatory cytokine therapies are not the only anti-inflammatory therapies for atherosclerosis and CVD management. Similar to the CANTOS trial, the LoDoCo trial was a first-of-its-kind prospective, observer-blinded clinical trial involving 532 participants diagnosed with coronary disease randomized to either a low-dose colchicine of 0.5 mg per day or non-colchicine group with a minimum two-year follow-up (158). However, unlike the CANTOS trial, which used a selective inhibitor of IL-1β, colchicine has broad-scale anti-inflammatory properties, most notably inhibiting neutrophil function (158). Of the LoDoCo participants, 93% were taking aspirin and clopidogrel, and 95% were taking statins. 282 participants were assigned to the colchicine group, while 250 were assigned to the non-colchicine group (158). Overall, the colchicine group had 10.7 percentage points fewer combined occurrences of acute coronary syndrome, out-of-hospital cardiac arrest, or non-cardioembolic ischemic stroke (primary outcomes) compared to the non-colchicine group (158). 15 of the 282 participants in the colchicine group experienced a primary outcome (5.3%) compared to 40 of the 250 patients in the non-colchicine group (16%) (158). However, this trial was open-labelled and moderate-scale, so these results needed further assessment (158). Following the LoDoCo trial, the COLCOT trial was conducted as a large-scale, randomized, parallel-arm, double-blind clinical trial involving 4,745 participants with an average follow-up period of 1.88 years (22.6 months) (159). The COLCOT trial recruited patients who had experienced myocardial infarction within the last 30 days. Of the 4,745 participants, 2,366 received 0.5 mg of colchicine daily, while 2,379 received a placebo. They assessed incidences of cardiovascular-related deaths, instances of resuscitated cardiac arrest, myocardial infarction strokes, and severe angina that ultimately required hospitalization (159). Overall, the colchicine group experienced 1.6 percentage points less of these cardiovascular outcomes than the placebo group. Of the 2,366 participants in the colchicine group, 5.5% experienced a cardiovascular outcome compared to the 7.1% in the placebo group (159).

Lastly, immune checkpoint inhibitors (ICI) are common mAbs used as anti-inflammatory therapies. As expanded on in section 1.2, immune checkpoints act as regulators of the immune response through T cell inhibition and activation. However, despite the success of ICI mAbs in cancer treatment, studies have found a significant link between ICI therapies and atherosclerosis (149). Interestingly, while cancer and atherosclerosis share similarities in their inflammatory-dependent pathophysiology, ICI mAbs, specifically CTLA-4 and PD-1–PD-L1 blocking antibodies, have been shown to increase cardiovascular events associated with atherosclerosis (160). Although, a 2013 study that increased CTLA-4 activity using abatacept in APOE 3-leiden mice found a reduction in the severity of atherosclerosis through a dramatic 78.1% decrease in arterial thickening (161), thus offering a different avenue for ICI therapy in atherosclerosis treatment. However, it is critical to recognize that these anti-inflammatory therapies have negligible effects on circulating lipoproteins, a major contributor to atherogenesis.

### 3.2 Lipid-lowering therapies and mAbs

Statin therapy has served as the pinnacle of lipid-lowering treatments in Western medicine for over four decades. Statins function by inhibiting the enzyme HMG-CoA reductase in the cholesterol biosynthesis pathway, thereby inducing the synthesis of LDLRs, which can then capture and reduce levels of circulating LDL (162). A plethora of research on statin application has demonstrated its success in LDL reduction. A 2010 meta-analysis including 26 randomized controlled trials with 169,138 participants revealed that a 39 mg/dL reduction in LDL resulted in a 22% decline in MACE over half a decade, independent of initial LDL levels and a 10% reduction in all-cause mortality across diverse clinical cohorts (163). Despite the well-documented lipid profile management of statins, cardiovascular events continue to occur in treated patients (164). This has been largely attributed to the contribution of dietary-derived remnant lipoproteins and Lp(a) to atherogenesis (165). In fact, the impact of statins in reducing Lp(a) remains controversial (166). A meta-analysis of several clinical trials demonstrated that they significantly increased plasma levels of this lipoprotein (167).

On the other hand, proprotein convertase subtilisin/kexin type 9 (PCSK9) inhibitors, which are lipid clearance agents that inhibit PCSK9-mediated degradation of LDLR, have been linked to reductions in circulating LDL, reduced myocardial infarction risk and overall decreases in mortality in some populations (13, 168). Prominent PCSK9 inhibitors include mAbs such as evolocumab, bococizumab, and alirocumab (169). Administration of solely evolocumab resulted in a 53% reduction in plasma LDL levels (168) with similar outcomes reported for bococizumab and alirocumab (151). When administered with statins, PCSK9 inhibitors have shown a pronounced reduction in LDL levels relative to statin monotherapy. The GLAGOV and ODYSSEY trials, large-scale randomized, double-blind clinical control studies, assessed the efficacy of evolocumab and alirocumab (with statins) in managing cardiovascular events (13, 169). The GLAGOV trial reported evolocumab therapy mediated plaque progression, and the combined regimen of evolocumab and statins induced regression of the proliferating atheroma (169). In the ODYSSEY trial, 80-88% of patients underwent statin treatment. The ODYSSEY trial found that combining alirocumab and statins reduced MACEs by 1.6 percentage points (9.5% for alirocumab *vs* 11.1% for placebo) and all-cause mortality by 0.6percentage points (3.5% for alirocumab *vs* 4.1% for placebo) compared to the placebo + statin treatment (169). A distinct randomized control trial by Pradhan et al. evaluated the combined regimen of bococizumab and statin therapy, involving 9,738 patients, and assessed on-treatment LDL levels 14 weeks post-intervention. The group reported a 60.5% reduction in LDL (170). However, they also acknowledged a significant correlation between patients with high hsCRP levels (>3 mg/L) and MACE. Moreover, even 14 weeks post-treatment, patients experienced residual risk associated with chronic inflammation (170). Pradhan et al. thus concluded that while PCSK9 and statin therapy reduce LDL levels and some MACE, they have minimal effects on inflammation (170).

Anti-PCSK9 mAbs can also reduce Lp(a) levels by 25~30%, at a 2:1 ratio relative to LDL (171). This effect appears to be mediated by a dual mechanism. When these drugs are administered as monotherapy, the decrease in the serum concentration of Lp(a) is associated with the inhibition of its synthesis while it has been suggested that, in combination with statins, anti-PCSK9 causes accelerated Lp(a) lipoprotein catabolism, potentially through increased LDLR activity (172). However, since statins (which increases the abundance of the hepatic LDLR), have limited impact on Lp(a) (167, 172), the exact role of LDLR in Lp(a) catabolism remains a matter of debate (172–174). A direct clinical benefit from the reduction in Lp(a) levels by anti-PCSK9 therapy has not yet been demonstrated (175).

Overall, lipid-lowering therapies, similar to anti-inflammatory approaches, are insufficient in fully modulating CVD risk among all populations, highlighting the need for emerging immunotherapies, including those directed at the arterial ECM.

### 3.3 Atherogenic lipoproteins and the need for combination therapies

Fasting LDL levels are often used as indicators and targets in the treatment and management of CVD, particularly atherosclerosis (176, 177). Fasting LDL levels are commonly measured in patients to determine their risk of overall CVD and prevent the onset of CVD. Interestingly, however, many patients continue to experience MACE despite reductions in LDL, known as residual risk. Additionally, non-fasting lipid levels have been proven to be an equal indicator of CVD risk compared to fasting lipid levels (178, 179). Non-fasting measurements are generally more representative of a patient’s lipid composition as most of the day is spent in the non-fasting state compared to the fasting state (38, 179–181). Due to the limitations regarding fasting LDL as both a target and assessment tool for CVD, research has shifted towards adopting more overarching approaches for CVD treatment. Recent advancements in our understanding of atherosclerosis have highlighted the critical role of remnant cholesterol in combination with LDL (177, 178, 182). Remnant cholesterol includes the cholesterol contained within remnant lipoproteins. These remnant lipoproteins come from triglyceride-rich lipoproteins such as liver-derived VLDL and intestinal chylomicron remnants (38, 180). Importantly, these remnant lipoproteins have been shown to play a critical role in atherogenesis. During the early stages of atherogenesis, both remnant lipoproteins and LDL infiltrate the inner tunica intima of the arterial wall, where they are digested by phagocytes, contributing to foam cell formation (38). Three extensive Copenhagen cohort studies previously illustrated the link between non-fasting remnant lipoprotein cholesterol and CVD (178, 183, 184).

Similarly, the Alberta tomorrow project (ATP) was a 2000 Canadian longitudinal cohort study that collected blood samples and health-related data. An analysis by Weaver et al. in 2023 using ATP data determined whether non-fasting remnant lipoprotein cholesterol could serve as a suitable indicator of CVD and future cardiovascular events, particularly in individuals with underlying health conditions like diabetes mellitus (177, 182). The group reported that non-fasting remnant lipoprotein cholesterol levels were significantly increased in individuals with CVD compared to the control group. However, this trend was not consistent for the group with diabetes and CVD (182). The diabetes + CVD group and the diabetes alone group had similar LDL levels. Furthermore, in 2023, a comprehensive large-scale investigation conducted by Navarese et al. used Mendelian randomization analysis techniques to determine the relationship between remnant lipoprotein cholesterol and the development of atherosclerosis-related CVD, specifically coronary artery disease, myocardial infarction, and stroke (185). The study used single nucleotide polymorphism associated with remnant lipoprotein cholesterol and LDL found on publicly available genome databases as representative variables for remnant cholesterol and LDL. The group additionally used data from various databases to create a participant pool of 958,434 people (185). Using the single nucleotide polymorphism for remnant cholesterol, the study found evidence of a strong relationship between remnant lipoprotein cholesterol levels and CVD risk. Each remnant lipoprotein cholesterol standard deviation (SD) increase was assigned a corresponding risk level expressed as an odds ratio (OR). For coronary artery disease, the group found that one SD increase in remnant lipoprotein cholesterol resulted in an OR of 1.51; for myocardial infarction, one SD increase resulted in an OR of 1.57; and for stroke, one SD increase resulted in an OR of 1.23 (185). Notably, this relationship between remnant lipoprotein cholesterol, coronary artery disease, myocardial infarction, and stroke was independent of LDL levels.

## 4 ChP3R99 mAb: emerging strategy for ApoB-containing lipoprotein retention

**Figure 3:**
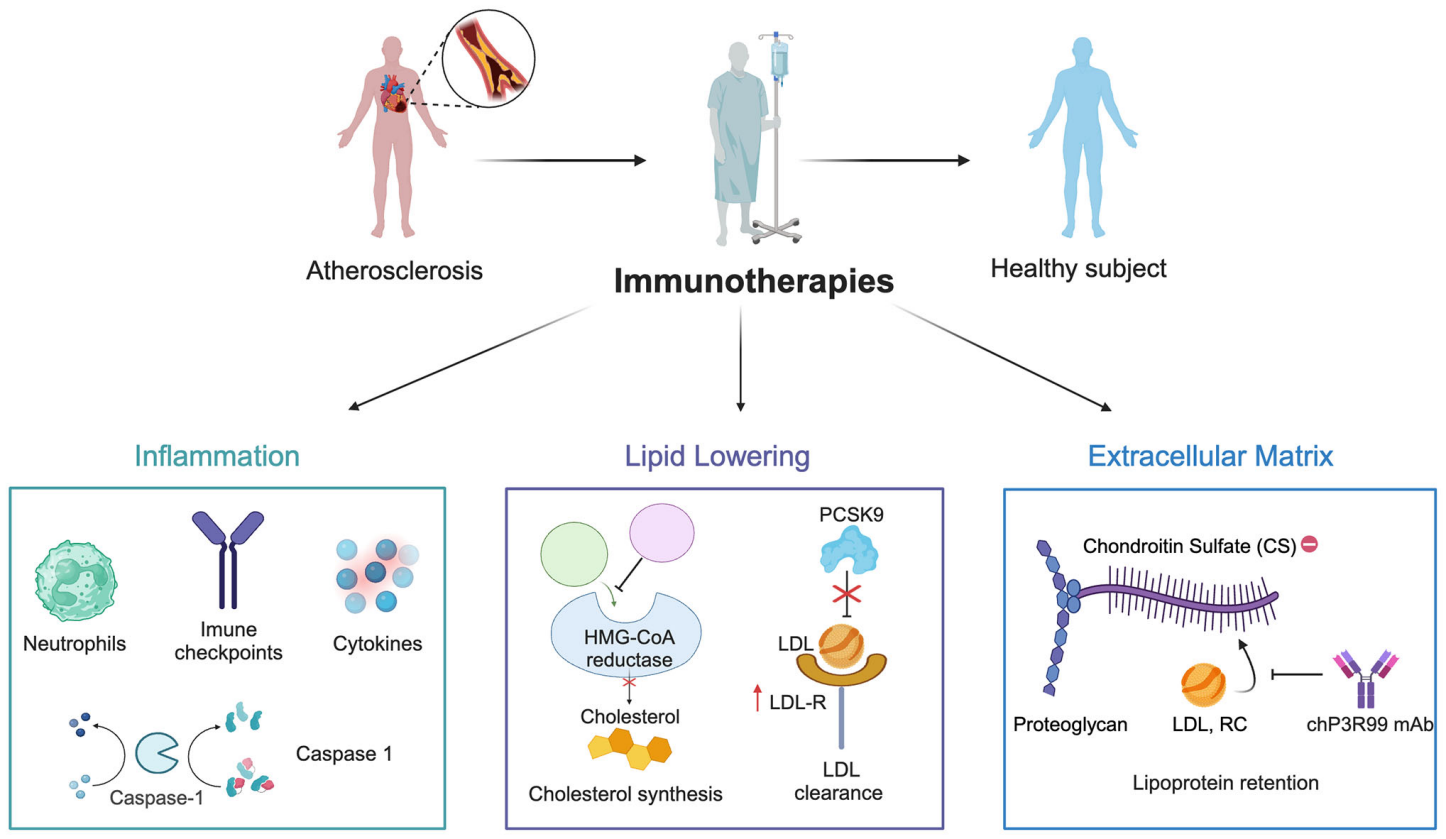
Mechanisms of immune-based therapies for atherosclerosis. The schematic illustrates current therapeutic approaches targeting inflammation (e.g. pro-inflammatory cytokines, immune checkpoints) and lipid lowering (e.g., HMG-CoA reductase and PCSK9 inhibition). The figure introduces emerging anti-extracellular matrix therapies aimed at reducing lipoprotein retention, chiefly the chP3R99 mAb, which targets sugar chains of arterial proteoglycans.

**Figure 4:**
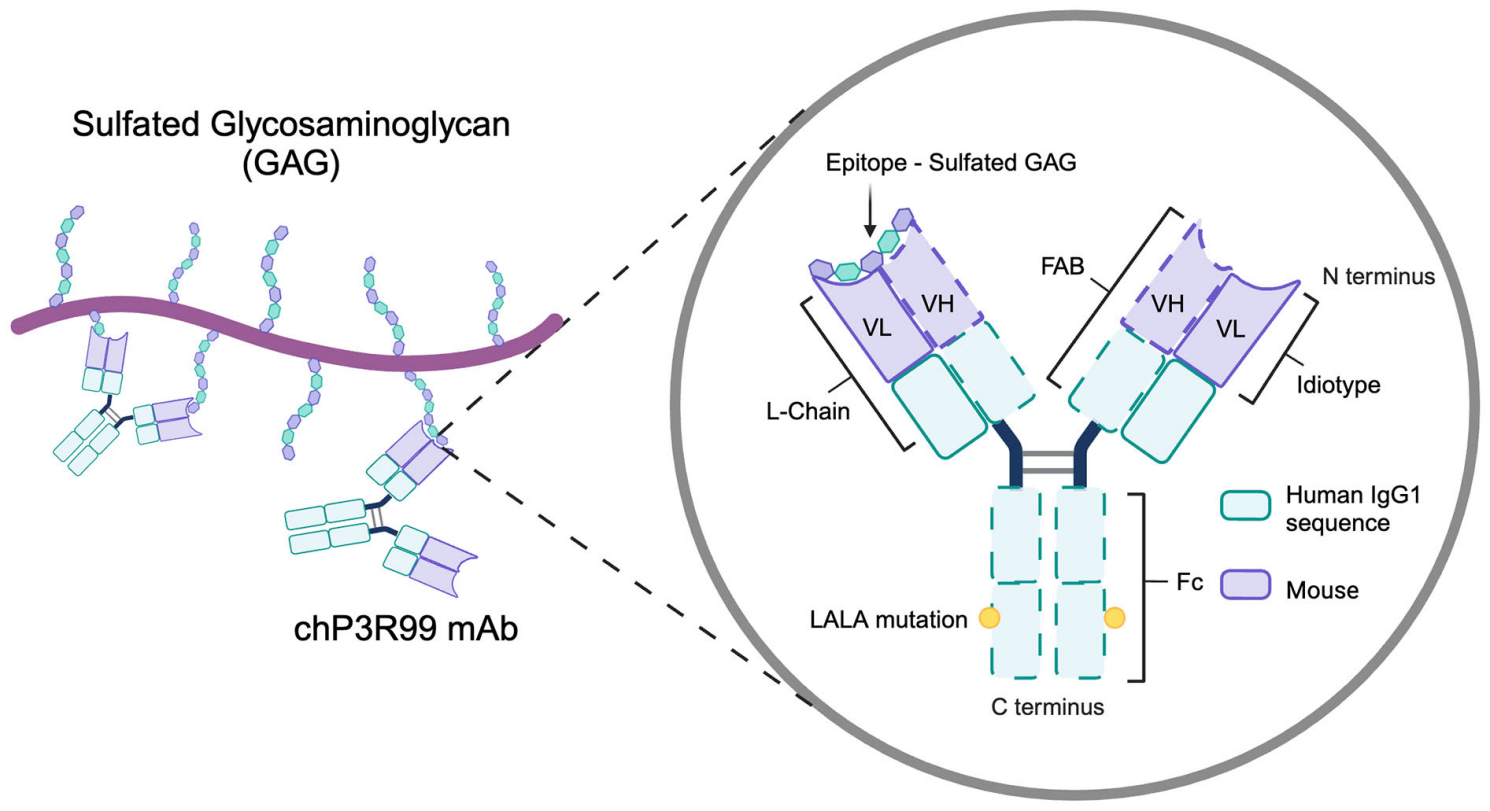
Structural and functional features of the chP3R99 mAb. The chP3R99 mAb is a mouse-to-human chimeric antibody designed to target sulfated glycosaminoglycans in the arterial extracellular matrix. It combines murine variable regions (VH and VL), essential for antigen recognition, with human IgG1 constant regions engineered with LALA mutations (L234A/L235A) to minimize inflammatory activation.

**Figure 5:**
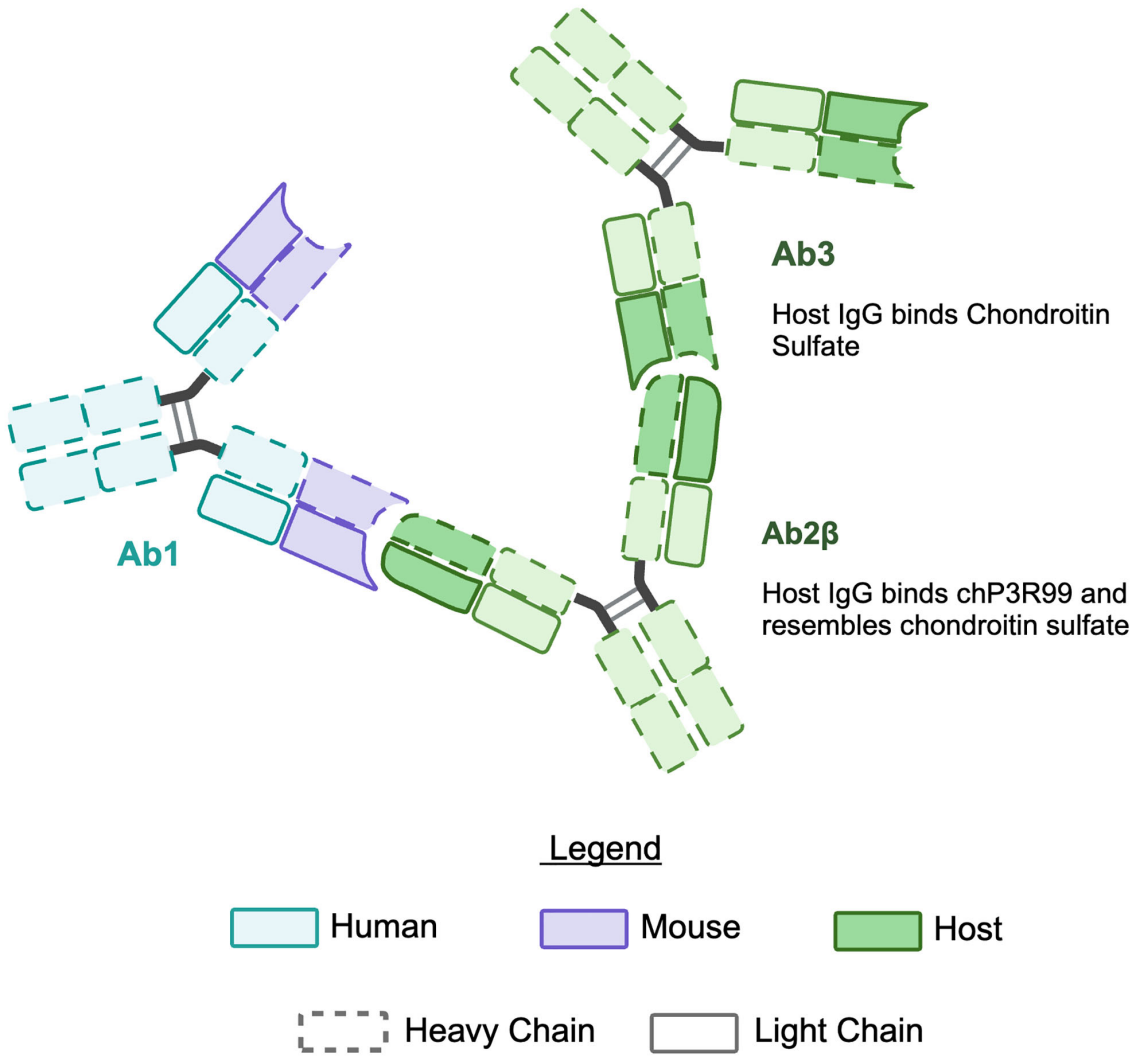
Illustration of the idiotypic cascade in response to chP3R99 mAb. Immunization with the chP3R99 mAb (Ab1) elicits an idiotypic cascade of endogenous antibodies in the host. The host produces anti-idiotypic antibodies (Ab2β) specific for the antigen-binding region of the Ab1. Subsequently, anti-anti-idiotypic antibodies (Ab3) are generated, which mimic Ab1 by binding chondroitin sulfate GAGs.

Advances in the understanding of atherogenesis have broadened the focus of mAbs for CVD, shifting beyond just targeting LDL and inflammatory cytokines to also include key vascular components (**Figure 3**) (186, 187). A notable example is the mAb chP3R99, which has emerged as a complementary approach to address ApoB-containing lipoprotein retention in the arterial wall (15). By binding to sulfated GAGs chains of arterial proteoglycans, chP3R99 is designed to interfere with lipoprotein retention, thereby mitigating subsequent oxidative stress, inflammation, and other processes central to plaque formation. Extensive preclinical studies using chP3R99 mAb have shown promising results in animal models of the disease, supporting its potential in preventing the early stages of atherosclerosis or halting its progression. Importantly, the therapeutic potential of chP3R99 extends beyond its passive blocking properties, offering a dual mechanism of action (1): as a passive therapy to acutely disrupt lipoprotein retention through direct binding to sulfated GAGs (**Figure 4**), and (2) as an idiotypic vaccine capable of inducing long-term protection via an anti-idiotypic cascade of antibodies induced in the host (**Figure 5**).

### 4.1 A historical perspective of the chP3R99 mAb’s development

The chP3R99 mAb was developed by the Centre for Molecular Immunology (CIM) in Havana, Cuba. This is a mouse-to-human chimeric antibody engineered to target the early stages of atherosclerosis within the arterial ECM (15). This antibody originated from the murine P3 mAb, an IgM first described by Vazquez et al. (1995), which was generated using the conventional hybridoma technique. Originally, P3 was intended to target N-glycolyl (NeuGc)–containing gangliosides as a potential tumor-specific immunotherapy (188). However, detailed characterization of its specificity revealed a strong reactivity toward sulfatides, demonstrating its ability to bind negatively charged epitopes on sugar moieties (188, 189). Notably, the polar head group of these glycolipids, composed of sulfated galactose, was identified as a critical structural element for this interaction. Further immunogenetic studies provided initial insights into P3’s binding specificity, suggesting that several basic aminoacidic residues in the variable regions, particularly those within the hypervariable loops, were important for antigen recognition (190).

To enhance its therapeutic potential, P3 was engineered into chP3, a chimeric mAb combining murine variable and human IgG1 constant regions (denoted by the prefix “ch”) that retained the specificity and main immunological properties of the parental mAb (191). Subsequently, site-specific single mutations of arginine residues at heavy chain complementarity determining regions 1 and 3 (HCDR1, HCDR3) of chP3 completely abolished antigen binding, confirming that these regions were crucial for the specificity of the mAb (192, 193). Afterwards, a mutant with a higher affinity for negatively charged sulfated glycolipids was designed. This chP3 mutant, termed chP3R99, was engineered by replacing the glutamic acid residue with an arginine at the 99^th^ position of the immunoglobulin HCDR3 (194). To further refine its therapeutic safety, the Fc region of chP3R99 was engineered with LALA mutations (L234A/L235A), disrupting Fcγ receptor and complement bindings to prevent undesired inflammatory activation while preserving its specificity (15). The current chP3R99-LALA variant is stably expressed in NS0 murine myeloma cells following transfection by electroporation (15, 194). In summary, chP3R99 mAb chimeric design retains the murine-derived variable regions (idiotype), critical for antigen recognition, while incorporating a human IgG1-LALA Fc portion (**Figure 4**).

### 4.2 Passive anti-atherogenic mechanism of the chP3R99 mAb

The rationale for evaluating chP3R99 mAb as a blocking agent in atherosclerosis emerged from two structural insights. First, the arginine-rich domains in its HCDRs mimic Site B of ApoB100, which mediates LDL retention via electrostatic interactions with arterial proteoglycans (190, 195). Second, sulfated *N*-acetyl galactosamine residues in CS-GAGs—critical for lipoprotein retention—share structural homology with the sulfated galactose head groups of sulfatides. Those similarities prompted the hypothesis that chP3R99 mAb could also recognize CS-GAGs. Hence, this engineered P3 mAb variant optimized for sulfated sugar epitope binding, could potentially compete with ApoB-containing lipoproteins for CS binding sites on arterial proteoglycans, thereby preventing subendothelial retention.

To test this hypothesis, Soto et al. (2012) characterized the reactivity of chP3R99 mAb to various GAGs. The study found that chP3R99 exhibited higher binding affinity to sulfated GAGs compared to the parental chP3 mAb, with preferential recognition to CS over other GAGs (15). Next, the team evaluated chP3R99’s efficacy in blocking LDL binding to CS. Solid-phase competition assays indicated that chP3R99 inhibited approximately ~70% of LDL binding to this GAG and further reduced ~80% of the LDL oxidation that is potentiated by LDL-CS interaction (15). *In vivo*, intravenous administration of chP3R99 in Sprague Dawley rats revealed a specific accumulation of the mAb within the aortic wall, associated with a significant decrease in LDL retention and subsequent oxidation 24 hours after LDL inoculation (15).

We have recently extended these findings to the arterial retention of both chylomicron remnants and LDL (the former being mediated by the Site B-Ib motif of ApoB48) (57, 140). *In vitro*, chP3R99 recognized CS and exhibited dose-dependent binding to ECM derived from rat VSMC. Solid-phase blocking experiments with equivalent concentrations of chP3R99 and ApoB48 demonstrated ~70% reduction of remnant binding to both CS and ECM. For LDL, comparable inhibition was observed for CS binding, while ~50% blocking was achieved for ECM interaction (140). The study further evaluated chP3R99 in obese insulin-resistant JCR: LA-cp rats, a model of vascular remodeling with increased production of CS proteoglycans and enhanced lipoprotein retention (45). Sequential perfusion of carotids from those rats at a physiological rate—first with chP3R99, followed by fluorescently labeled chylomicron remnants—demonstrated dose-dependent inhibition of remnant retention *in situ*. Notably, these particles displaced only ~35% of the chP3R99 bound to carotid tissue, while cholesterol deposition in the arterial wall was drastically reduced by the treatment by ~80%, underscoring the mAb’s efficacy for chylomicron remnants (140).

In a separate competitive perfusion experiment, carotid arteries were exposed to a preparation containing equivalent particle numbers of LDL and remnants (normalized by ApoB100/ApoB48). Here, insulin-resistant rats exhibited 3.6-fold higher LDL retention and 2.8-fold higher remnant retention compared to lean controls. Despite remnants’ lower particle retention, their cholesterol deposition was 6-fold greater than LDL, aligned with their larger size and a higher cholesterol content per particle. In this setting, chP3R99 reduced LDL retention more effectively by particle count while its overall proportional impact on cholesterol deposition was markedly greater for remnants, highlighting the relevance of targeting both classes of lipoproteins (140).

While chP3R99’s efficacy against Lp(a) has not been tested yet, its specificity for sulfated GAGs suggests potential to reduce ApoB100-mediated retention of Lp(a) by CS proteoglycans (196). However, Lp(a) retention also comprises Apo(a)-specific mechanisms involving other ECM components, including binding to fibronectin (197). These additional mechanisms could limit chP3R99’s efficacy against Lp(a) compared to other ApoB-containing lipoproteins whose retention relies solely on proteoglycan interactions. Definitive evaluation requires competitive binding assays with purified Lp(a) and *in vivo* validation in *LPA*-transgenic models to dissect chP3R99’s therapeutic potential for this high-risk lipoprotein.

The previous findings support chP3R99 as a passive therapy for atherosclerosis, relying on direct binding to arterial proteoglycans over secondary immune mechanisms. This strategy is particularly relevant for secondary prevention in patients with advanced plaques requiring acute stabilization, enabled by its Fc-silenced design to minimize inflammatory risks (198). However, its potential in primary prevention—such as high-risk populations with familial hypercholesterolemia or elevated Lp(a)—requires further exploration, given its mechanistic focus on lipoprotein retention. Unlike immunization, which induces long-term protection, passive administration provides immediate, transient blockade at high dose.

### 4.3 Vaccine-like effects of the chP3R99 mAb

In addition to its blocking properties, chP3R99 exhibits vaccine-like effects mediated by its unique idiotype, which stimulates a robust anti-idiotypic antibody cascade across species (15, 140, 142). This immunogenic trait is inherited from P3, a murine antibody that paradoxically demonstrated high intrinsic immunogenicity in syngeneic BALB/c mice, even without adjuvants or carrier proteins (199). The immunodominance of P3’s idiotype is driven by germline-encoded T-cell epitopes within its murine variable regions, enabling MHC class II presentation by APCs and ultimately the induction of an anti-idiotypic cascade (190, 200). Remarkably, this immunodominance persists in chP3 (191, 192) and chP3R99 (142) despite their chimeric design, where the murine idiotype represents only ~30% of the antibody’s structure, whereas the human IgG1 Fc portion is expected to be immunodominant in mice (15).

This phenomenon aligns with the principles of Jerne’s idiotypic network theory (1974) (201), wherein an antibody (Ab1) induces anti-idiotypic antibodies (Ab2) specific for its idiotype. A subset of these Ab2 (termed Ab2β) structurally mimics the antigen recognized by Ab1, acting as an “internal image” of the antigen—in this case, the sulfated sugar epitopes targeted by chP3R99. This cascade is further amplified through the production of Ab3 (anti-Ab2), which recapitulate the specificity of the original Ab1, thereby enhancing therapeutic efficacy (**Figure 5**). As a result, both chP3R99 and the induced Ab3 recognize CS, preventing lipoprotein retention and subsequent plaque formation (140, 142).

The vaccine-like effects of chP3R99 mAb are further characterized by dose-dependent immunogenicity and broad applicability across age and gender (202). This response typically reaches a plateau after four mAb administrations (140, 202). Preclinical studies in ApoE mice demonstrated that subcutaneous administration of the mAb induces anti-CS IgG1 antibodies, a Th2-associated subclass in mice, targeting lipoprotein retention without eliciting proinflammatory responses, a critical safety feature for atherosclerosis therapy (202). Notably, mice exhibited comparable anti-CS antibody titers after immunization, regardless of age or sex. A 4-fold increase in chP3R99 dose enhanced both the magnitude and kinetics of the idiotypic cascade, generating significantly higher Ab2 and Ab3 responses while reducing reactivity to the human Fc domain (202).

While chP3R99’s murine idiotype drives robust responses in mice, its immunogenicity in other animal models is subjected to cross-species compatibility of T-cell epitopes and MHC binding affinity. Therefore, original T-cell epitopes may lose immunogenicity in non-murine systems, or model-specific epitopes may emerge (203). However, the idiotype’s foreign nature ensures sustained immunogenicity across diverse animal models, though the human constant regions could potentially shift immunodominance toward the Fc portion. To date, chP3R99 has consistently demonstrated immunogenicity in mice, rats, and rabbits, inducing anti-CS antibodies capable of blocking lipoprotein retention in all tested models (15, 140, 142). Unpublished results have further validated its immunogenicity and idiotype immunodominance in outbred NMRI mice and Landrace pigs, underscoring its efficacy in genetically diverse populations.

On the other hand, translating chP3R99’s immunogenicity into humans requires careful consideration of three factors (1): the HLA polymorphism (204), (2) the need of T-cell epitopes to enable APC antigen presentation (203)—in this case solely restricted to the idiotype, and (3) antibody engineering trade-offs (205). Indeed, the murine idiotype of chP3R99 provides structural diversity for T-cell epitopes, increasing the likelihood of compatibility with different HLA alleles to ensure antigen presentation (204, 206). Although a fully murine format may enhance its immunogenicity, there is a risk of shifting immunodominance toward the Fc region, promoting anti-isotype responses over idiotype-specific immunity critical for vaccination. Conversely, full humanization may alter or disrupt T-cell epitopes within the variable region abolishing anti-idiotype responses (203). Therefore, chP3R99’s chimeric design aims to circumvent these drawbacks: the murine idiotype likely retains its immunogenicity in humans (via preserved T-cell epitopes) (205, 207) while the human IgG1-LALA Fc improve safety, extends half-life for passive immunotherapy, and avoids Fc-driven inflammation (15, 194).

### 4.4 Preclinical evidence of anti-atherogenic effects of chP3R99 mAb

The chP3R99 mAb has shown compelling anti-atherogenic effects in preclinical models, targeting both early and advanced stages of atherosclerosis. Proof-of-concept was first established in an acute atherosclerosis model using NZW rabbits, where the disease was induced via 8-day intravenous Lipofundin 20% lipid emulsion (208). Prophylactic immunization with chP3R99 (100 µg SC weekly, 3 total doses) prevented atheromatous lesions in 57% of animals, with the remaining rabbits exhibiting only minor intimal thickening (15). Notably, immunization with the parental chP3 mAb lacked this protective effect. Treated rabbits also showed reduced lipid peroxidation and preserved endothelial nitric oxide bioavailability, demonstrating the vaccination capacity to mitigate oxidative stress and vascular dysfunction (15). This preventative effect was further validated in a chronic atherosclerosis model using ApoE**^−/−^** mice fed a high-fat, high-cholesterol diet. Biweekly/weekly chP3R99 immunization (50 µg SC, 6 doses), starting at 6 weeks of age, reduced aortic lesion area by 40–43% by week 18 (142). In both models, the protection was associated with host-derived anti-CS antibodies (Ab3) that blocked LDL-CS binding *in vitro*, supporting the hypothesis of the induction of a protective idiotypic cascade. No lipid-lowering effects were observed in those studies, emphasizing that the mechanism of action relies on antibody-mediated inhibition of LDL retention rather than lipid metabolic modulation (15, 142).

Subsequent studies investigated the therapeutic effects of chP3R99 in established atherosclerosis using the preclinical models described above. In rabbits, weekly subcutaneous administration of chP3R99 (100 µg, 5 weeks) following Lipofundin-induced lesion formation reduced atherosclerotic plaque burden, characterized by a significantly decreased intimal thickening (209). In a parallel study, 18-week-old ApoE−/− mice were placed on a high-fat, high-cholesterol diet for 14 weeks before vaccination and maintained on this atherogenic diet throughout the experiment. In this model, weekly subcutaneous administration of 50 µg of the vaccine over six weeks effectively halted atherosclerotic lesion progression, even in the presence of persistent hyperlipidemia (210). In both models, chP3R99 vaccination reduced aortic oxidative stress, evidenced by decreased levels of malondialdehyde and advanced oxidation protein products, while enhancing antioxidant capacity and nitric oxide bioavailability (209, 210).

Importantly, both male and female ApoE mice fed a hypercholesterolemic diet exhibited comparable reductions in atherosclerotic lesions (~35–40%) when immunized with chP3R99 (202). Further dose-escalation studies in this model revealed that higher doses of chP3R99 (200 µg/week) significantly improved efficacy, achieving a 62% reduction in atherosclerotic lesions compared to a 40% reduction at the dose of 50 µg/week (202). At higher doses, it is plausible that the combined passive effect of the mAb and the induction of anti-CS antibodies operate synergistically to enhance the overall antiatherogenic effect. Supporting this hypothesis, Brito et al. (2017) demonstrated that chP3R99 conjugated to FITC preferentially accumulated within aortic lesions in ApoE mice *in vivo* (211), while arterial accumulation was similarly observed in rats (15, 140). Additionally, recent studies in insulin-resistant JCR: LA-cp rats fed a lipid-balanced hypercholesterolemic diet showed that anti-CS antibodies induced by immunization specifically accumulated in arterial regions, leading to reduced retention of LDL and chylomicron remnants in carotid arteries (140). In ApoE mice with stablished plaques (11 weeks of disease progression), chP3R99 limited lesion expansion at the aortic level and reduced inflammatory infiltrates highlighting its therapeutic potential. These findings support further investigation into chP3R99’s role in mitigating advanced disease progression and its promise as a candidate for secondary prevention strategies in high-risk patients. Its ability to reduce advanced disease progression positions chP3R99 as a promising candidate for secondary prevention strategies in high-risk patients.

Beyond its therapeutic applications, chP3R99 also exhibits significant diagnostic potential. In rabbits with early atherosclerosis lesions induced by Lipofundin 20%, immunoscintigraphy using radiolabeled ^99m^Tc-chP3R99 demonstrated specific accumulation within carotid lesions compared to healthy vessels (141). Histological studies and biodistribution analyses further confirmed a six-fold higher accumulation in atherosclerosis-prone regions of the aorta in diseased animals compared to controls (141). Similarly, *in vivo* immunofluorescence studies in ApoE mice demonstrated that FITC-labeled chP3R99 preferentially accumulated in atherosclerotic lesions within the aorta compared to a control mAb (142). These findings highlight the ability of chP3R99 to specifically target atherosclerotic lesions, supporting its potential for non-invasive plaque imaging or site-specific therapeutic delivery.

While sulfated GAGs are physiologically relevant and widely expressed across various tissues, chP3R99 has demonstrated high vascular specificity, a crucial attribute for minimizing off-target effects. Biodistribution studies support that chP3R99 exhibits high selectivity for proteoglycans derived from VSMCs within atherosclerotic lesions (140), with limited accumulation in non-vascular tissues (141). Preclinical evaluations in mice, rats, and rabbits revealed no adverse effects on lipoprotein metabolism or signs of toxicity (15, 140–142). Specifically, in insulin-resistant and wild-type rats, neither passive administration of chP3R99 nor immunization with this mAb affected lipid or glucose metabolism, hepatic or renal function, or blood cell indices (140). Importantly, the Fc-silenced chP3R99-LALA variant further eliminated Fc-mediated risks, reinforcing its safety profile and translational potential for clinical applications (198, 212).

In summary, chP3R99 offers a multifaceted therapeutic approach with potential applications in both primary and secondary prevention of atherosclerotic disease. For primary prevention, particularly in high-risk populations such as those with familial hypercholesterolemia or elevated Lp(a), immunization strategies may offer long-term protection by inducing a sustained anti-atherogenic antibody response (213). Conversely, in secondary prevention scenarios involving patients with advanced plaques requiring acute intervention (214), passive administration of chP3R99 could provide immediate benefits by directly blocking lipoprotein retention. Future clinical trials should prioritize cohorts with high atherogenic burden and those unresponsive to conventional treatments to evaluate chP3R99´s efficacy in addressing refractory lipoprotein retention and its potential to improve outcomes in patients with established CVD.

## 5 Conclusion

Despite advancements in lipid-lowering and anti-inflammatory therapies, residual cardiovascular risk persists in atherosclerosis. Existing therapies for CVD, while effective at reducing LDL cholesterol, exhibit limited efficacy against Lp(a) and dietary-derived remnant lipoproteins, the latter contributing significantly to atherogenesis in chronic disease(s). Crucially, most interventions prioritize systemic risk factors over targeting the arterial ECM, where ApoB-containing lipoproteins bind sulfated GAGs chains on proteoglycans, triggering oxidative stress and inflammation. Therefore, the chP3R99 mAb represents a transformative shift in the therapeutic landscape. By targeting arterial lipoprotein retention, it disrupts atherogenesis through dual mechanisms (1): direct blockade of ApoB-GAGs interactions (passive therapy) and (2) induction of anti-idiotypic antibodies that sustain long-term protection against proteoglycan-mediated retention (idiotypic vaccine). Preclinical studies demonstrate that chP3R99 prevents atherosclerosis initiation, arrests disease progression, and exerts efficacy even in advanced lesions, consolidating sulfated GAGs as pivotal mediators across all stages of atherogenesis and highlighting the mAb’s broad therapeutic applicability. While translational validation in humans and efficacy against Lp(a) remain essential, this ECM-centric approach bridges a critical gap in current therapies, offering a strategy to reduce residual risk and redefine atherosclerosis management.

## References

[1] Williams KJ Tabas I. The response-to-retention hypothesis of early atherogenesis. Arterioscler Thromb Vasc Biol. (1995) 15:551–61. doi: 10.1161/01.ATV.15.5.551 PMC2924812 7749869

[2] Bäck M Yurdagul A Tabas I Öörni K Kovanen PT. Inflammation and its resolution in atherosclerosis: mediators and therapeutic opportunities. Nat Rev Cardiol. (2019) 16:389–406. doi: 10.1038/s41569-019-0169-2 30846875 PMC6727648

[3] Yurdagul A Jr. Finney AC Woolard MD Orr AW. The arterial microenvironment: the where and why of atherosclerosis. Biochem J. (2016) 473:1281–95. doi: 10.1042/BJ20150844 PMC5410666 27208212

[4] Fogelstrand P Boren J. Retention of atherogenic lipoproteins in the artery wall and its role in atherogenesis. Nutr Metab Cardiovasc Dis. (2012) 22:1–7. doi: 10.1016/j.numecd.2011.09.007 22176921

[5] Tannock LR King VL. Proteoglycan mediated lipoprotein retention: a mechanism of diabetic atherosclerosis. Rev Endocr Metab Disord. (2008) 9:289–300. doi: 10.1007/s11154-008-9078-0 18584330

[6] Lundberg AM Hansson GK. Innate immune signals in atherosclerosis. Clin Immunol. (2010) 134:5–24. doi: 10.1016/j.clim.2009.07.016 19740706

[7] Lu RM Hwang YC Liu IJ Lee CC Tsai HZ Li HJ. Development of therapeutic antibodies for the treatment of diseases. J BioMed Sci. (2020) 27:1. doi: 10.1186/s12929-019-0592-z 31894001 PMC6939334

[8] Makover ME Shapiro MD Toth PP. There is urgent need to treat atherosclerotic cardiovascular disease risk earlier, more intensively, and with greater precision: A review of current practice and recommendations for improved effectiveness. Am J Prev Cardiol. (2022) 12:100371. doi: 10.1016/j.ajpc.2022.100371 36124049 PMC9482082

[9] Sirtori CR. The pharmacology of statins. Pharmacol Res. (2014) 88:3–11. doi: 10.1016/j.phrs.2014.03.002 24657242

[10] Rikhi R Shapiro MD. Newer and emerging LDL-C lowering agents and implications for ASCVD residual risk. J Clin Med. (2022) 11:4611. doi: 10.3390/jcm11154611 35956226 PMC9369522

[11] Shapiro MD Fazio S. From lipids to inflammation: new approaches to reducing atherosclerotic risk. Circ Res. (2016) 118:732–49. doi: 10.1161/CIRCRESAHA.115.306471 26892970

[12] Bermudez V Rojas-Quintero J Velasco M. The quest for immunotherapy in atherosclerosis: CANTOS study, interleukin-1beta and vascular inflammation. J Thorac Dis. (2018) 10:64–9. doi: 10.21037/jtd.2017.12.47 PMC5863198 29600023

[13] Kim K Ginsberg HN Choi SH. New, novel lipid-lowering agents for reducing cardiovascular risk: beyond statins. Diabetes Metab J. (2022) 46:517–32. doi: 10.4093/dmj.2022.0198 PMC9353557 35929170

[14] Ait-Oufella H Libby P Tedgui A. Antibody-based immunotherapy targeting cytokines and atherothrombotic cardiovascular diseases. Arch Cardiovasc Dis. (2020) 113:5–8. doi: 10.1016/j.acvd.2019.11.001 31917124

[15] Soto Y Acosta E Delgado L Perez A Falcon V Becquer MA. Antiatherosclerotic effect of an antibody that binds to extracellular matrix glycosaminoglycans. Arterioscler Thromb Vasc Biol. (2012) 32:595–604. doi: 10.1161/ATVBAHA.111.238659 22267481

[16] Herrington W Lacey B Sherliker P Armitage J Lewington S. Epidemiology of atherosclerosis and the potential to reduce the global burden of atherothrombotic disease. Circ Res. (2016) 118:535–46. doi: 10.1161/CIRCRESAHA.115.307611 26892956

[17] Ruparelia N Chai JT Fisher EA Choudhury RP. Inflammatory processes in cardiovascular disease: a route to targeted therapies. Nat Rev Cardiol. (2017) 14:133–44. doi: 10.1038/nrcardio.2016.185 PMC5525550 27905474

[18] Ibanez B Fernandez-Ortiz A Fernandez-Friera L Garcia-Lunar I Andres V Fuster V. Progression of early subclinical atherosclerosis (PESA) study: JACC focus seminar 7/8. J Am Coll Cardiol. (2021) 78:156–79. doi: 10.1016/j.jacc.2021.05.011 34238438

[19] Libby P. Molecular and cellular mechanisms of the thrombotic complications of atherosclerosis. J Lipid Res. (2009) 50 Suppl:S352–7. doi: 10.1194/jlr.R800099-JLR200 PMC2674742 19096046

[20] Song P Fang Z Wang H Cai Y Rahimi K Zhu Y. Global and regional prevalence, burden, and risk factors for carotid atherosclerosis: a systematic review, meta-analysis, and modelling study. Lancet Glob Health. (2020) 8:e721–e9. doi: 10.1016/S2214-109X(20)30117-0 32353319

[21] Shah P Bajaj S Virk H Bikkina M Shamoon F. Rapid progression of coronary atherosclerosis: A review. Thrombosis. (2015) 2015:634983. doi: 10.1155/2015/634983 26823982 PMC4707354

[22] Knoflach M Kiechl S Penz D Zangerle A Schmidauer C Rossmann A. Cardiovascular risk factors and atherosclerosis in young women: atherosclerosis risk factors in female youngsters (ARFY study). Stroke. (2009) 40:1063–9. doi: 10.1161/STROKEAHA.108.525675 19211497

[23] Poznyak AV Sadykhov NK Kartuesov AG Borisov EE Melnichenko AA Grechko AV. Hypertension as a risk factor for atherosclerosis: Cardiovascular risk assessment. Front Cardiovasc Med. (2022) 9:959285. doi: 10.3389/fcvm.2022.959285 36072873 PMC9441708

[24] Zarate A Manuel-Apolinar L Saucedo R Hernandez-Valencia M Basurto L. Hypercholesterolemia as a risk factor for cardiovascular disease: current controversial therapeutic management. Arch Med Res. (2016) 47:491–5. doi: 10.1016/j.arcmed.2016.11.009 28262189

[25] Ross R. Atherosclerosis–an inflammatory disease. N Engl J Med. (1999) 340:115–26. doi: 10.1056/NEJM199901143400207 9887164

[26] Ross R Glomset J Harker L. Response to injury and atherogenesis. Am J Pathol. (1977) 86:675–84. PMC2032127 842616

[27] Boren J Williams KJ. The central role of arterial retention of cholesterol-rich apolipoprotein-B-containing lipoproteins in the pathogenesis of atherosclerosis: a triumph of simplicity. Curr Opin Lipidol. (2016) 27:473–83. doi: 10.1097/MOL.0000000000000330 27472409

[28] Williams KJ Tabas I Fisher EA. How an artery heals. Circ Res. (2015) 117:909–13. doi: 10.1161/CIRCRESAHA.115.307609 PMC4663458 26541678

[29] Simionescu N Simionescu M. Cellular interactions of lipoproteins with the vascular endothelium: endocytosis and transcytosis. Targeted Diagn Ther. (1991) 5:45–95. doi: 10.1201/9780203748831-3 1797171

[30] Vasile E Simionescu M Simionescu N. Visualization of the binding, endocytosis, and transcytosis of low-density lipoprotein in the arterial endothelium in situ. J Cell Biol. (1983) 96:1677–89. doi: 10.1083/jcb.96.6.1677 PMC2112465 6853599

[31] Armstrong SM Sugiyama MG Fung KY Gao Y Wang C Levy AS. A novel assay uncovers an unexpected role for SR-BI in LDL transcytosis. Cardiovasc Res. (2015) 108:268–77. doi: 10.1093/cvr/cvv218 PMC4614686 26334034

[32] Kraehling JR Chidlow JH Rajagopal C Sugiyama MG Fowler JW Lee MY. Genome-wide RNAi screen reveals ALK1 mediates LDL uptake and transcytosis in endothelial cells. Nat Commun. (2016) 7:13516. doi: 10.1038/ncomms13516 27869117 PMC5121336

[33] Huang L Chambliss KL Gao X Yuhanna IS Behling-Kelly E Bergaya S. SR-B1 drives endothelial cell LDL transcytosis via DOCK4 to promote atherosclerosis. Nature. (2019) 569:565–9. doi: 10.1038/s41586-019-1140-4 PMC6631346 31019307

[34] Lee S Schleer H Park H Jang E Boyer M Tao B. Genetic or therapeutic neutralization of ALK1 reduces LDL transcytosis and atherosclerosis in mice. Nat Cardiovasc Res. (2023) 2:438–48. doi: 10.1038/s44161-023-00266-2 PMC11358031 39196046

[35] Cabodevilla AG Tang S Lee S Mullick AE Aleman JO Hussain MM. Eruptive xanthoma model reveals endothelial cells internalize and metabolize chylomicrons, leading to extravascular triglyceride accumulation. J Clin Invest. (2021) 131. doi: 10.1172/JCI145800 PMC8203467 34128469

[36] Ginsberg HN Packard CJ Chapman MJ Boren J Aguilar-Salinas CA Averna M. Triglyceride-rich lipoproteins and their remnants: metabolic insights, role in atherosclerotic cardiovascular disease, and emerging therapeutic strategies-a consensus statement from the European Atherosclerosis Society. Eur Heart J. (2021) 42:4791–806. doi: 10.1093/eurheartj/ehab551 PMC8670783 34472586

[37] Proctor SD Vine DF Mamo JC. Arterial permeability and efflux of apolipoprotein B-containing lipoproteins assessed by in *situ* perfusion and three-dimensional quantitative confocal microscopy. Arterioscler Thromb Vasc Biol. (2004) 24:2162–7. doi: 10.1161/01.ATV.0000143859.75035.5a 15345509

[38] Proctor SD Mamo JC. Retention of fluorescent-labelled chylomicron remnants within the intima of the arterial wall–evidence that plaque cholesterol may be derived from post-prandial lipoproteins. Eur J Clin Invest. (1998) 28:497–503. doi: 10.1046/j.1365-2362.1998.00317.x 9693943

[39] Kronenberg F Mora S Stroes ESG Ference BA Arsenault BJ Berglund L. Lipoprotein(a) in atherosclerotic cardiovascular disease and aortic stenosis: a European Atherosclerosis Society consensus statement. Eur Heart J. (2022) 43:3925–46. doi: 10.1093/eurheartj/ehac361 PMC9639807 36036785

[40] Boren J Packard CJ Binder CJ. Apolipoprotein B-containing lipoproteins in atherogenesis. Nat Rev Cardiol. (2025) 2025:1–15. doi: 10.1038/s41569-024-01111-0 39743565

[41] Boutagy NE Gamez-Mendez A Fowler JW Zhang H Chaube BK Esplugues E. Dynamic metabolism of endothelial triglycerides protects against atherosclerosis in mice. J Clin Invest. (2024) 134. doi: 10.1172/JCI170453 PMC10866653 38175710

[42] Jaffe IZ Karumanchi SA. Lipid droplets in the endothelium: The missing link between metabolic syndrome and cardiovascular disease? J Clin Invest. (2024) 134. doi: 10.1172/JCI176347 PMC10866645 38357921

[43] Kim B Zhao W Tang SY Levin MG Ibrahim A Yang Y. Endothelial lipid droplets suppress eNOS to link high fat consumption to blood pressure elevation. J Clin Invest. (2023) 133. doi: 10.1172/JCI173160 PMC10721151 37824206

[44] Proctor SD Vine DF Mamo JC. Arterial retention of apolipoprotein B(48)- and B(100)-containing lipoproteins in atherogenesis. Curr Opin Lipidol. (2002) 13:461–70. doi: 10.1097/00041433-200210000-00001 12352009

[45] Mangat R Warnakula S Borthwick F Hassanali Z Uwiera RR Russell JC. Arterial retention of remnant lipoproteins ex vivo is increased in insulin resistance because of increased arterial biglycan and production of cholesterol-rich atherogenic particles that can be improved by ezetimibe in the JCR: LA-cp rat. J Am Heart Assoc. (2012) 1:e003434. doi: 10.1161/JAHA.112.003434 23316299 PMC3541624

[46] Proctor SD Mamo JC. Intimal retention of cholesterol derived from apolipoprotein B100- and apolipoprotein B48-containing lipoproteins in carotid arteries of Watanabe heritab le hyperlipidemic rabbits. Arterioscler Thromb Vasc Biol. (2003) 23:1595–600. doi: 10.1161/01.ATV.0000084638.14534.0A 12842838

[47] Tabas I Garcia-Cardena G Owens GK. Recent insights into the cellular biology of atherosclerosis. J Cell Biol. (2015) 209:13–22. doi: 10.1083/jcb.201412052 25869663 PMC4395483

[48] Mangat R Su JW Lambert JE Clandinin MT Wang Y Uwiera RR. Increased risk of cardiovascular disease in Type 1 diabetes: arterial exposure to remnant lipoproteins leads to enhanced deposition of cholesterol and binding to glycated extracellular matrix proteoglycans. Diabetes Med. (2011) 28:61–72. doi: 10.1111/j.1464-5491.2010.03138.x 21166847

[49] Camejo G Hurt-Camejo E Wiklund O Bondjers G. Association of apo B lipoproteins with arterial proteoglycans: pathological significance and molecular basis. Atherosclerosis. (1998) 139:205–22. doi: 10.1016/S0021-9150(98)00107-5 9712326

[50] Skalen K Gustafsson M Rydberg EK Hulten LM Wiklund O Innerarity TL. Subendothelial retention of atherogenic lipoproteins in early atherosclerosis. Nature. (2002) 417:750–4. doi: 10.1038/nature00804 12066187

[51] Hurt-Camejo E Camejo G. ApoB-100 lipoprotein complex formation with intima proteoglycans as a cause of atherosclerosis and its possible ex vivo evaluation as a disease biomarker. J Cardiovasc Dev Dis. (2018) 5:36. doi: 10.20944/preprints201806.0239.v1 29966388 PMC6162553

[52] Pulai JI Neuman RJ Groenewegen AW Wu J Schonfeld G. Genetic heterogeneity in familial hypobetalipoproteinemia: linkage and non-linkage to the apoB gene in Caucasian families. Am J Med Genet. (1998) 76:79–86. doi: 10.1002/(SICI)1096-8628(19980226)76:1<79::AID-AJMG15>3.0.CO;2-M 9508071

[53] Law SW Lackner KJ Hospattankar AV Anchors JM Sakaguchi AY Naylor SL. Human apolipoprotein B-100: cloning, analysis of liver mRNA, and assignment of the gene to chromosome 2. Proc Natl Acad Sci U S A. (1985) 82:8340–4. doi: 10.1073/pnas.82.24.8340 PMC390911 3001697

[54] Fisher EA Ginsberg HN. Complexity in the secretory pathway: the assembly and secretion of apolipoprotein B-containing lipoproteins. J Biol Chem. (2002) 277:17377–80. doi: 10.1074/jbc.R100068200 12006608

[55] Chan L Apolipoprotein B. the major protein component of triglyceride-rich and low density lipoproteins. J Biol Chem. (1992) 267:25621–4. doi: 10.1016/S0021-9258(18)35646-1 1464582

[56] Boren J Lee I Zhu W Arnold K Taylor S Innerarity TL. Identification of the low density lipoprotein receptor-binding site in apolipoprotein B100 and the modulation of its binding activity by the carboxyl terminus in familial defective apo-B100. J Clin Invest. (1998) 101:1084–93. doi: 10.1172/JCI1847 PMC508660 9486979

[57] Flood C Gustafsson M Richardson PE Harvey SC Segrest JP Boren J. Identification of the proteoglycan binding site in apolipoprotein B48. J Biol Chem. (2002) 277:32228–33. doi: 10.1074/jbc.M204053200 12070165

[58] Proctor SD Wang M Vine DF Raggi P. Predictive utility of remnant cholesterol in atherosclerotic cardiovascular disease. Curr Opin Cardiol. (2024) 39:300–7. doi: 10.1097/HCO.0000000000001140 38456429

[59] Back M Weber C Lutgens E. Regulation of atherosclerotic plaque inflammation. J Intern Med. (2015) 278:462–82. doi: 10.1111/joim.2015.278.issue-5 25823439

[60] Ensan S Li A Besla R Degousee N Cosme J Roufaiel M. Self-renewing resident arterial macrophages arise from embryonic CX3CR1(+) precursors and circulating monocytes immediately after birth. Nat Immunol. (2016) 17:159–68. doi: 10.1038/ni.3343 26642357

[61] Poznyak AV Nikiforov NG Starodubova AV Popkova TV Orekhov AN. Macrophages and foam cells: brief overview of their role, linkage, and targeting potential in atherosclerosis. Biomedicines. (2021) 9:1221. doi: 10.3390/biomedicines9091221 34572406 PMC8468383

[62] Poznyak AV Wu WK Melnichenko AA Wetzker R Sukhorukov V Markin AM. Signaling Pathways Key Genes Involved Regul foam Cell Formation Atherosclerosis. Cells (2020) 9:584. doi: 10.3390/cells9030584 32121535 PMC7140394

[63] Theofilis P Oikonomou E Tsioufis K Tousoulis D. The role of macrophages in atherosclerosis: pathophysiologic mechanisms and treatment considerations. Int J Mol Sci. (2023) 24:9568. doi: 10.3390/ijms24119568 37298518 PMC10253295

[64] Williams JW Zaitsev K Kim KW Ivanov S Saunders BT Schrank PR. Limited proliferation capacity of aortic intima resident macrophages requires monocyte recruitment for atherosclerotic plaque progression. Nat Immunol. (2020) 21:1194–204. doi: 10.1038/s41590-020-0768-4 PMC7502558 32895539

[65] Medrano-Bosch M Simon-Codina B Jimenez W Edelman ER Melgar-Lesmes P. Monocyte-endothelial cell interactions in vascular and tissue remodeling. Front Immunol. (2023) 14:1196033. doi: 10.3389/fimmu.2023.1196033 37483594 PMC10360188

[66] Libby P Lichtman AH Hansson GK. Immune effector mechanisms implicated in atherosclerosis: from mice to humans. Immunity. (2013) 38:1092–104. doi: 10.1016/j.immuni.2013.06.009 PMC3764500 23809160

[67] Susser LI Rayner KJ. Through the layers: how macrophages drive atherosclerosis across the vessel wall. J Clin Invest. (2022) 132. doi: 10.1172/JCI157011 PMC9057606 35499077

[68] Bennett MR Sinha S Owens GK. Vascular smooth muscle cells in atherosclerosis. Circ Res. (2016) 118:692–702. doi: 10.1161/CIRCRESAHA.115.306361 26892967 PMC4762053

[69] Jebari-Benslaiman S Galicia-Garcia U Larrea-Sebal A Olaetxea JR Alloza I Vandenbroeck K. Pathophysiology of Atherosclerosis. Int J Mol Sci. (2022) 23:3346. doi: 10.3390/ijms23063346 35328769 PMC8954705

[70] Little PJ Ballinger ML Burch ML Osman N. Biosynthesis of natural and hyperelongated chondroitin sulfate glycosaminoglycans: new insights into an elusive process. Open Biochem J. (2008) 2:135–42. doi: 10.2174/1874091X00802010135 PMC2627520 19238187

[71] Kumarapperuma H Chia ZJ Malapitan SM Wight TN Little PJ Kamato D. Response to retention hypothesis as a source of targets for arterial wall-directed therapies to prevent atherosclerosis: A critical review. Atherosclerosis. (2024) 397:118552. doi: 10.1016/j.atherosclerosis.2024.118552 39180958

[72] Wang Y Dubland JA Allahverdian S Asonye E Sahin B Jaw JE. Smooth muscle cells contribute the majority of foam cells in apoE (Apolipoprotein E)-deficient mouse atherosclerosis. Arterioscler Thromb Vasc Biol. (2019) 39:876–87. doi: 10.1161/ATVBAHA.119.312434 PMC6482082 30786740

[73] Pan H Xue C Auerbach BJ Fan J Bashore AC Cui J. Single-cell genomics reveals a novel cell state during smooth muscle cell phenotypic switching and potential therapeutic targets for atherosclerosis in mouse and human. Circulation. (2020) 142:2060–75. doi: 10.1161/CIRCULATIONAHA.120.048378 PMC8104264 32962412

[74] Tellides G Pober JS. Inflammatory and immune responses in the arterial media. Circ Res. (2015) 116:312–22. doi: 10.1161/CIRCRESAHA.116.301312 25593276

[75] Olejarz W Lacheta D Kubiak-Tomaszewska G. Matrix metalloproteinases as biomarkers of atherosclerotic plaque instability. Int J Mol Sci. (2020) 21:3946. doi: 10.3390/ijms21113946 32486345 PMC7313469

[76] Poznyak AV Nikiforov NG Markin AM Kashirskikh DA Myasoedova VA Gerasimova EV. Overview of oxLDL and its impact on cardiovascular health: focus on atherosclerosis. Front Pharmacol. (2020) 11:613780. doi: 10.3389/fphar.2020.613780 33510639 PMC7836017

[77] Herrero-Fernandez B Gomez-Bris R Somovilla-Crespo B Gonzalez-Granado JM. Immunobiology of atherosclerosis: a complex net of interactions. Int J Mol Sci. (2019) 20:5293. doi: 10.3390/ijms20215293 31653058 PMC6862594

[78] Mushenkova NV Nikiforov NG Melnichenko AA Kalmykov V Shakhpazyan NK Orekhova VA. Functional phenotypes of intraplaque macrophages and their distinct roles in atherosclerosis development and atheroinflammation. Biomedicines. (2022) 10:452. doi: 10.3390/biomedicines10020452 35203661 PMC8962399

[79] Wu T Peng Y Yan S Li N Chen Y Lan T. Andrographolide ameliorates atherosclerosis by suppressing pro-inflammation and ROS generation-mediated foam cell formation. Inflammation. (2018) 41:1681–9. doi: 10.1007/s10753-018-0812-9 29948505

[80] Ganesan R Henkels KM Wrenshall LE Kanaho Y Di Paolo G Frohman MA. Oxidized LDL phagocytosis during foam cell formation in atherosclerotic plaques relies on a PLD2-CD36 functional interdependence. J Leukoc Biol. (2018) 103:867–83. doi: 10.1002/JLB.2A1017-407RR PMC5951301 29656494

[81] Gao LN Zhou X Lu YR Li K Gao S Yu CQ. Dan-Lou Prescription Inhibits Foam Cell Formation Induced by ox-LDL via the TLR4/NF-kappaB and PPARgamma Signaling Pathways. Front Physiol. (2018) 9:590. doi: 10.3389/fphys.2018.00590 29896109 PMC5987004

[82] Papac-Milicevic N Busch CJ Binder CJ. Malondialdehyde epitopes as targets of immunity and the implications for atherosclerosis. Adv Immunol. (2016) 131:1–59. doi: 10.1016/bs.ai.2016.02.001 27235680 PMC5892703

[83] Greaves DR Gordon S. The macrophage scavenger receptor at 30 years of age: current knowledge and future challenges. J Lipid Res. (2009) 50 Suppl:S282–6. doi: 10.1194/jlr.R800066-JLR200 PMC2674758 19074372

[84] He Y Hara H Núñez G. Mechanism and regulation of NLRP3 inflammasome activation. Trends Biochem Sci. (2016) 41:1012–21. doi: 10.1016/j.tibs.2016.09.002 PMC5123939 27669650

[85] Hui Y Ricciotti E Crichton I Yu Z Wang D Stubbe J. Targeted deletions of cyclooxygenase-2 and atherogenesis in mice. Circulation. (2010) 121:2654–60. doi: 10.1161/CIRCULATIONAHA.109.910687 PMC2909762 20530000

[86] Lendeckel U Venz S Wolke C. Macrophages: shapes and functions. ChemTexts. (2022) 8:12. doi: 10.1007/s40828-022-00163-4 35287314 PMC8907910

[87] Boyle JJ. Macrophage activation in atherosclerosis: pathogenesis and pharmacology of plaque rupture. Curr Vasc Pharmacol. (2005) 3:63–8. doi: 10.2174/1570161052773861 15638783

[88] Steinberg D Witztum JL. Oxidized low-density lipoprotein and atherosclerosis. Arterioscler Thromb Vasc Biol. (2010) 30:2311–6. doi: 10.1161/ATVBAHA.108.179697 21084697

[89] Roy P Orecchioni M Ley K. How the immune system shapes atherosclerosis: roles of innate and adaptive immunity. Nat Rev Immunol. (2022) 22:251–65. doi: 10.1038/s41577-021-00584-1 PMC10111155 34389841

[90] Glass CK Witztum JL. Atherosclerosis. the road ahead. Cell. (2001) 104:503–16. 10.1016/s0092-8674(01)00238-0 10.1016/s0092-8674(01)00238-0 11239408

[91] Zhou X Caligiuri G Hamsten A Lefvert AK Hansson GK. LDL immunization induces T-cell-dependent antibody formation and protection against atherosclerosis. Arterioscler Thromb Vasc Biol. (2001) 21:108–14. doi: 10.1161/01.ATV.21.1.108 11145941

[92] Sun J Sukhova GK Wolters PJ Yang M Kitamoto S Libby P. Mast cells promote atherosclerosis by releasing proinflammatory cytokines. Nat Med. (2007) 13:719–24. doi: 10.1038/nm1601 17546038

[93] Subramanian M Tabas I. Dendritic cells in atherosclerosis. Semin Immunopathol. (2014) 36:93–102. doi: 10.1007/s00281-013-0400-x 24196454 PMC3946524

[94] Taams LS van Eden W Wauben MH. Antigen presentation by T cells versus professional antigen-presenting cells (APC): differential consequences for T cell activation and subsequent T cell-APC interactions. Eur J Immunol. (1999) 29:1543–50. doi: 10.1002/(SICI)1521-4141(199905)29:05<1543::AID-IMMU1543>3.0.CO;2-R 10359108

[95] Banchereau J Briere F Caux C Davoust J Lebecque S Liu YJ. Immunobiology of dendritic cells. Annu Rev Immunol. (2000) 18:767–811. doi: 10.1146/annurev.immunol.18.1.767 10837075

[96] Zernecke A. Dendritic cells in atherosclerosis: evidence in mice and humans. Arterioscler Thromb Vasc Biol. (2015) 35:763–70. doi: 10.1161/ATVBAHA.114.303566 25675999

[97] Bousso P. T-cell activation by dendritic cells in the lymph node: lessons from the movies. Nat Rev Immunol. (2008) 8:675–84. doi: 10.1038/nri2379 19172690

[98] Bobryshev YV Ivanova EA Chistiakov DA Nikiforov NG Orekhov AN. Macrophages and their role in atherosclerosis: pathophysiology and transcriptome analysis. BioMed Res Int. (2016) 2016:9582430. doi: 10.1155/2016/9582430 27493969 PMC4967433

[99] Shaw MK Tse KY Zhao X Welch K Eitzman DT Thipparthi RR. T-cells specific for a self-peptide of apoB-100 exacerbate aortic atheroma in murine atherosclerosis. Front Immunol. (2017) 8:95. doi: 10.3389/fimmu.2017.00095 28280493 PMC5322236

[100] Jones PW Mallat Z Nus M. T-cell/B-cell interactions in atherosclerosis. Arterioscler Thromb Vasc Biol. (2024) 44:1502–11. doi: 10.1161/ATVBAHA.124.319845 PMC11208060 38813700

[101] Robertson AK Hansson GK. cells in atherogenesis: for better or for worse T Arterioscler Thromb Vasc Biol. (2006) 26:2421–32. doi: 10.1161/01.ATV.0000245830.29764.84 16973967

[102] Stemme S Faber B Holm J Wiklund O Witztum JL Hansson GK. T lymphocytes from human atherosclerotic plaques recognize oxidized low density lipoprotein. Proc Natl Acad Sci U S A. (1995) 92:3893–7. doi: 10.1073/pnas.92.9.3893 PMC42068 7732003

[103] Palinski W Miller E Witztum JL. Immunization of low density lipoprotein (LDL) receptor-deficient rabbits with homologous malondialdehyde-modified LDL reduces atherogenesis. Proc Natl Acad Sci U S A. (1995) 92:821–5. doi: 10.1073/pnas.92.3.821 PMC42712 7846059

[104] Ameli S Hultgardh-Nilsson A Regnstrom J Calara F Yano J Cercek B. Effect of immunization with homologous LDL and oxidized LDL on early atherosclerosis in hypercholesterolemic rabbits. Arterioscler Thromb Vasc Biol. (1996) 16:1074–9. doi: 10.1161/01.ATV.16.8.1074 8696949

[105] Saigusa R Roy P Freuchet A Gulati R Ghosheh Y Suthahar SSA. Single cell transcriptomics and TCR reconstruction reveal CD4 T cell response to MHC-II-restricted APOB epitope in human cardiovascular disease. Nat Cardiovasc Res. (2022) 1:462–75. doi: 10.1038/s44161-022-00063-3 PMC9383695 35990517

[106] Kimura T Kobiyama K Winkels H Tse K Miller J Vassallo M. Regulatory CD4(+) T cells recognize major histocompatibility complex class II molecule-restricted peptide epitopes of apolipoprotein B. Circulation. (2018) 138:1130–43. doi: 10.1161/CIRCULATIONAHA.117.031420 PMC6160361 29588316

[107] Kimura T Tse K McArdle S Gerhardt T Miller J Mikulski Z. Atheroprotective vaccination with MHC-II-restricted ApoB peptides induces peritoneal IL-10-producing CD4 T cells. Am J Physiol Heart Circ Physiol. (2017) 312:H781–H90. doi: 10.1152/ajpheart.00798.2016 PMC5407161 28087520

[108] Tse K Gonen A Sidney J Ouyang H Witztum JL Sette A. Atheroprotective vaccination with MHC-II restricted peptides from apoB-100. Front Immunol. (2013) 4:493. doi: 10.3389/fimmu.2013.00493 24416033 PMC3873602

[109] Marchini T Hansen S Wolf D. ApoB-specific CD4(+) T cells in mouse and human atherosclerosis. Cells. (2021) 10:446. doi: 10.3390/cells10020446 33669769 PMC7922692

[110] Hansson GK Libby P. The immune response in atherosclerosis: a double-edged sword. Nat Rev Immunol. (2006) 6:508–19. doi: 10.1038/nri1882 16778830

[111] Libby P Ridker PM Hansson GK. Progress and challenges in translating the biology of atherosclerosis. Nature. (2011) 473:317–25. doi: 10.1038/nature10146 21593864

[112] Saigusa R Winkels H Ley K. T cell subsets and functions in atherosclerosis. Nat Rev Cardiol. (2020) 17:387–401. doi: 10.1038/s41569-020-0352-5 32203286 PMC7872210

[113] Frostegard J Ulfgren AK Nyberg P Hedin U Swedenborg J Andersson U. Cytokine expression in advanced human atherosclerotic plaques: dominance of pro-inflammatory (Th1) and macrophage-stimulating cytokines. Atherosclerosis. (1999) 145:33–43. doi: 10.1016/S0021-9150(99)00011-8 10428293

[114] Profumo E Buttari B Saso L Capoano R Salvati B Rigano R. T lymphocyte autoreactivity in inflammatory mechanisms regulating atherosclerosis. ScientificWorldJournal. (2012) 2012:157534. doi: 10.1100/2012/157534 23304078 PMC3529860

[115] Hansson GK Berne GP. Atherosclerosis and the immune system. Acta Paediatr Suppl. (2004) 93(446):63–9. doi: 10.1111/j.1651-2227.2004.tb00241.x 15702672

[116] 116. Buono C Binder CJ Stavrakis G Witztum JL Glimcher LH Lichtman AH. -bet deficiency reduces atherosclerosis and alters plaque antigen-specific immune responses. T Proc Natl Acad Sci U S A. (2005) 102:1596–601. doi: 10.1073/pnas.0409015102 PMC547865 15665085

[117] Ait-Oufella H Salomon BL Potteaux S Robertson AK Gourdy P Zoll J. Natural regulatory T cells control the development of atherosclerosis in mice. Nat Med. (2006) 12:178–80. doi: 10.1038/nm1343 16462800

[118] Klingenberg R Gerdes N Badeau RM Gistera A Strodthoff D Ketelhuth DF. Depletion of FOXP3+ regulatory T cells promotes hypercholesterolemia and atherosclerosis. J Clin Invest. (2013) 123:1323–34. doi: 10.1172/JCI63891 PMC3582120 23426179

[119] Tupin E Nicoletti A Elhage R Rudling M Ljunggren HG Hansson GK. CD1d-dependent activation of NKT cells aggravates atherosclerosis. J Exp Med. (2004) 199:417–22. doi: 10.1084/jem.20030997 PMC2211791 14744994

[120] Taleb S Romain M Ramkhelawon B Uyttenhove C Pasterkamp G Herbin O. Loss of SOCS3 expression in T cells reveals a regulatory role for interleukin-17 in atherosclerosis. J Exp Med. (2009) 206:2067–77. doi: 10.1084/jem.20090545 PMC2757872 19737863

[121] Gapin L Matsuda JL Surh CD Kronenberg M. NKT cells derive from double-positive thymocytes that are positively selected by CD1d. Nat Immunol. (2001) 2:971–8. doi: 10.1038/ni710 11550008

[122] Kyaw T Winship A Tay C Kanellakis P Hosseini H Cao A. Cytotoxic and proinflammatory CD8+ T lymphocytes promote development of vulnerable atherosclerotic plaques in apoE-deficient mice. Circulation. (2013) 127:1028–39. doi: 10.1161/CIRCULATIONAHA.112.001347 23395974

[123] Schafer S Zernecke A. CD8(+) T cells in atherosclerosis. Cells. (2020) 10:27. doi: 10.3390/cells10010037 33383733 PMC7823404

[124] He C Kim HI Park J Guo J Huang W. The role of immune cells in different stages of atherosclerosis. Int J Med Sci. (2024) 21:1129–43. doi: 10.7150/ijms.94570 PMC11103388 38774746

[125] Cochain C Koch M Chaudhari SM Busch M Pelisek J Boon L. CD8+ T cells regulate monopoiesis and circulating ly6C-high monocyte levels in atherosclerosis in mice. Circ Res. (2015) 117:244–53. doi: 10.1161/CIRCRESAHA.117.304611 25991812

[126] Dimayuga PC Zhao X Yano J Lio WM Zhou J Mihailovic PM. Identification of apoB-100 peptide-specific CD8+ T cells in atherosclerosis. J Am Heart Assoc. (2017) 6. doi: 10.1161/JAHA.116.005318 PMC5586274 28711866

[127] Ramshaw AL Parums DV. Immunohistochemical characterization of inflammatory cells associated with advanced atherosclerosis. Histopathology. (1990) 17:543–52. doi: 10.1111/j.1365-2559.1990.tb00794.x 2076887

[128] Yin C Mohanta SK Srikakulapu P Weber C Habenicht AJ. Artery tertiary lymphoid organs: powerhouses of atherosclerosis immunity. Front Immunol. (2016) 7:387. doi: 10.3389/fimmu.2016.00387 27777573 PMC5056324

[129] Binder CJ Silverman GJ. Natural antibodies and the autoimmunity of atherosclerosis. Springer Semin Immunopathol. (2005) 26:385–404. doi: 10.1007/s00281-004-0185-z 15609021

[130] Hansson GK Hermansson A. The immune system in atherosclerosis. Nat Immunol. (2011) 12:204–12. doi: 10.1038/ni.2001 21321594

[131] Sage AP Tsiantoulas D Binder CJ Mallat Z. The role of B cells in atherosclerosis. Nat Rev Cardiol. (2019) 16:180–96. doi: 10.1038/s41569-018-0106-9 30410107

[132] Kyaw T Tay C Khan A Dumouchel V Cao A To K. Conventional B2 B cell depletion ameliorates whereas its adoptive transfer aggravates atherosclerosis. J Immunol. (2010) 185:4410–9. doi: 10.4049/jimmunol.1000033 20817865

[133] Sage AP Mallat Z. Multiple potential roles for B cells in atherosclerosis. Ann Med. (2014) 46:297–303. doi: 10.3109/07853890.2014.900272 24813455

[134] Kyaw T Tay C Krishnamurthi S Kanellakis P Agrotis A Tipping P. B1a B lymphocytes are atheroprotective by secreting natural IgM that increases IgM deposits and reduces necrotic cores in atherosclerotic lesions. Circ Res. (2011) 109:830–40. doi: 10.1161/CIRCRESAHA.111.248542 21868694

[135] Ait-Oufella H Herbin O Bouaziz JD Binder CJ Uyttenhove C Laurans L. B cell depletion reduces the development of atherosclerosis in mice. J Exp Med. (2010) 207:1579–87. doi: 10.1084/jem.20100155 PMC2916123 20603314

[136] Tsimikas S Brilakis ES Lennon RJ Miller ER Witztum JL McConnell JP. Relationship of IgG and IgM autoantibodies to oxidized low density lipoprotein with coronary artery disease and cardiovascular events. J Lipid Res. (2007) 48:425–33. doi: 10.1194/jlr.M600361-JLR200 17093289

[137] Soto Y Conde H Aroche R Brito V Luaces P Nasiff A. Autoantibodies to oxidized low density lipoprotein in relation with coronary artery disease. Hum Antibodies. (2009) 18:109–17. doi: 10.3233/HAB-2009-0202 19729805

[138] Li J Ley K. Lymphocyte migration into atherosclerotic plaque. Arterioscler Thromb Vasc Biol. (2015) 35:40–9. doi: 10.1161/ATVBAHA.114.303227 PMC4429868 25301842

[139] Koltsova EK Garcia Z Chodaczek G Landau M McArdle S Scott SR. Dynamic T cell-APC interactions sustain chronic inflammation in atherosclerosis. J Clin Invest. (2012) 122:3114–26. doi: 10.1172/JCI61758 PMC3428082 22886300

[140] Soto Y Hernandez A Sarduy R Brito V Marleau S Vine DF. Monoclonal antibody chP3R99 reduces subendothelial retention of atherogenic lipoproteins in insulin-resistant rats: acute treatment versus long-term protection as an idiotypic vaccine for atherosclerosis. J Am Heart Assoc. (2024) 13:e032419. doi: 10.1161/JAHA.123.032419 38934863 PMC11255714

[141] Soto Y Mesa N Alfonso Y Perez A Batlle F Grinan T. Targeting arterial wall sulfated glycosaminoglycans in rabbit atherosclerosis with a mouse/human chimeric antibody. MAbs. (2014) 6:1340–6. doi: 10.4161/mabs.29970 PMC4622498 25517318

[142] Brito V Mellal K Portelance SG Perez A Soto Y deBlois D. Induction of anti-anti-idiotype antibodies against sulfated glycosaminoglycans reduces atherosclerosis in apolipoprotein E-deficient mice. Arterioscler Thromb Vasc Biol. (2012) 32:2847–54. doi: 10.1161/ATVBAHA.112.300444 23087361

[143] Stierschneider A Wiesner C. Shedding light on the molecular and regulatory mechanisms of TLR4 signaling in endothelial cells under physiological and inflamed conditions. Front Immunol. (2023) 14:1264889. doi: 10.3389/fimmu.2023.1264889 38077393 PMC10704247

[144] Hellenthal KEM Brabenec L Wagner NM. Regulation and dysregulation of endothelial permeability during systemic inflammation. Cells. (2022) 11:1935. doi: 10.3390/cells11121935 35741064 PMC9221661

[145] Claesson-Welsh L Dejana E McDonald DM. Permeability of the endothelial barrier: identifying and reconciling controversies. Trends Mol Med. (2021) 27:314–31. doi: 10.1016/j.molmed.2020.11.006 PMC8005435 33309601

[146] Chen L Qu H Liu B Chen BC Yang Z Shi DZ. Low or oscillatory shear stress and endothelial permeability in atherosclerosis. Front Physiol. (2024) 15:1432719. doi: 10.3389/fphys.2024.1432719 39314624 PMC11417040

[147] Poston RN. Atherosclerosis: integration of its pathogenesis as a self-perpetuating propagating inflammation: a review. Cardiovasc Endocrinol Metab. (2019) 8:51–61. doi: 10.1097/XCE.0000000000000172 31588428 PMC6738649

[148] Ley K Gerdes N Winkels H. ATVB distinguished scientist award: how costimulatory and coinhibitory pathways shape atherosclerosis. Arterioscler Thromb Vasc Biol. (2017) 37:764–77. doi: 10.1161/ATVBAHA.117.308611 PMC5424816 28360089

[149] Yousif LI Tanja AA de Boer RA Teske AJ Meijers WC. The role of immune checkpoints in cardiovascular disease. Front Pharmacol. (2022) 13:989431. doi: 10.3389/fphar.2022.989431 36263134 PMC9574006

[150] Chen L Flies DB. Molecular mechanisms of T cell co-stimulation and co-inhibition. Nat Rev Immunol. (2013) 13:227–42. doi: 10.1038/nri3405 PMC3786574 23470321

[151] Ridker PM Everett BM Thuren T MacFadyen JG Chang WH Ballantyne C. Antiinflammatory therapy with canakinumab for atherosclerotic disease. N Engl J Med. (2017) 377:1119–31. doi: 10.1056/NEJMoa1707914 28845751

[152] Pedro-Botet J Climent E Benaiges D. Atherosclerosis and inflammation. New Ther approaches. Med Clin (Barc). (2020) 155:256–62. doi: 10.1016/j.medcli.2020.04.024 32571617

[153] Tousoulis D Oikonomou E Economou EK Crea F Kaski JC. Inflammatory cytokines in atherosclerosis: current therapeutic approaches. Eur Heart J. (2016) 37:1723–32. doi: 10.1093/eurheartj/ehv759 26843277

[154] Coll RC Robertson AA Chae JJ Higgins SC Munoz-Planillo R Inserra MC. A small-molecule inhibitor of the NLRP3 inflammasome for the treatment of inflammatory diseases. Nat Med. (2015) 21:248–55. doi: 10.1038/nm.3806 PMC4392179 25686105

[155] Netea MG Joosten LA. Inflammasome inhibition: putting out the fire. Cell Metab. (2015) 21:513–4. doi: 10.1016/j.cmet.2015.03.012 25863243

[156] Zeng W Wu D Sun Y Suo Y Yu Q Zeng M. The selective NLRP3 inhibitor MCC950 hinders atherosclerosis development by attenuating inflammation and pyroptosis in macrophages. Sci Rep. (2021) 11:19305. doi: 10.1038/s41598-021-98437-3 34588488 PMC8481539

[157] Youm YH Nguyen KY Grant RW Goldberg EL Bodogai M Kim D. The ketone metabolite beta-hydroxybutyrate blocks NLRP3 inflammasome-mediated inflammatory disease. Nat Med. (2015) 21:263–9. doi: 10.1038/nm.3804 PMC4352123 25686106

[158] Nidorf SM Eikelboom JW Budgeon CA Thompson PL. Low-dose colchicine for secondary prevention of cardiovascular disease. J Am Coll Cardiol. (2013) 61:404–10. doi: 10.1016/j.jacc.2012.10.027 23265346

[159] Tardif JC Kouz S Waters DD Bertrand OF Diaz R Maggioni AP. Efficacy and safety of low-dose colchicine after myocardial infarction. N Engl J Med. (2019) 381:2497–505. doi: 10.1056/NEJMoa1912388 31733140

[160] Suero-Abreu GA Zanni MV Neilan TG. Atherosclerosis with immune checkpoint inhibitor therapy: evidence, diagnosis, and management: JACC: cardioOncology state-of-the-art review. JACC CardioOncol. (2022) 4:598–615. doi: 10.1016/j.jaccao.2022.11.011 36636438 PMC9830225

[161] Ewing MM Karper JC Abdul S de Jong RC Peters HA de Vries MR. T-cell co-stimulation by CD28-CD80/86 and its negative regulator CTLA-4 strongly influence accelerated atherosclerosis development. Int J Cardiol. (2013) 168:1965–74. doi: 10.1016/j.ijcard.2012.12.085 23351788

[162] McFarland AJ Anoopkumar-Dukie S Arora DS Grant GD McDermott CM Perkins AV. Molecular mechanisms underlying the effects of statins in the central nervous system. Int J Mol Sci. (2014) 15:20607–37. doi: 10.3390/ijms151120607 PMC4264186 25391045

[163] Cholesterol Treatment Trialists C Mihaylova B Emberson J Blackwell L Keech A Simes J. The effects of lowering LDL cholesterol with statin therapy in people at low risk of vascular disease: meta-analysis of individual data from 27 randomised trials. Lancet. (2012) 380:581–90. doi: 10.1016/S0140-6736(12)60367-5 PMC3437972 22607822

[164] Libby P. The forgotten majority: unfinished business in cardiovascular risk reduction. J Am Coll Cardiol. (2005) 46:1225–8. doi: 10.1016/j.jacc.2005.07.006 16198835

[165] Linton MF Yancey PG Davies SS Jerome WG Linton EF Song WL. The role of lipids and lipoproteins in atherosclerosis. In: Feingold KR Ahmed SF, editors. Endotext. South dartmouth (MA) (2019).

[166] Lippi G Targher G. Optimal therapy for reduction of lipoprotein(a). J Clin Pharm Ther. (2012) 37:1–3. doi: 10.1111/j.1365-2710.2011.01244.x 22276844

[167] Tsimikas S Gordts P Nora C Yeang C Witztum JL. Statin therapy increases lipoprotein(a) levels. Eur Heart J. (2020) 41:2275–84. doi: 10.1093/eurheartj/ehz310 31111151

[168] Farnier M. PCSK9: From discovery to therapeutic applications. Arch Cardiovasc Dis. (2014) 107:58–66. doi: 10.1016/j.acvd.2013.10.007 24373748

[169] Rosenson RS Hegele RA Fazio S Cannon CP. The evolving future of PCSK9 inhibitors. J Am Coll Cardiol. (2018) 72:314–29. doi: 10.1016/j.jacc.2018.04.054 30012326

[170] Pradhan AD Aday AW Rose LM Ridker PM. Residual inflammatory risk on treatment with PCSK9 inhibition and statin therapy. Circulation. (2018) 138:141–9. doi: 10.1161/CIRCULATIONAHA.118.034645 PMC8108606 29716940

[171] Gaudet D Kereiakes DJ McKenney JM Roth EM Hanotin C Gipe D. Effect of alirocumab, a monoclonal proprotein convertase subtilisin/kexin 9 antibody, on lipoprotein(a) concentrations (a pooled analysis of 150 mg every two weeks dosing from phase 2 trials). Am J Cardiol. (2014) 114:711–5. doi: 10.1016/j.amjcard.2014.05.060 25060413

[172] Watts GF Chan DC Somaratne R Wasserman SM Scott R Marcovina SM. Controlled study of the effect of proprotein convertase subtilisin-kexin type 9 inhibition with evolocumab on lipoprotein(a) particle kinetics. Eur Heart J. (2018) 39:2577–85. doi: 10.1093/eurheartj/ehy122 29566128

[173] Reyes-Soffer G Pavlyha M Ngai C Thomas T Holleran S Ramakrishnan R. Effects of PCSK9 inhibition with alirocumab on lipoprotein metabolism in healthy humans. Circulation. (2017) 135:352–62. doi: 10.1161/CIRCULATIONAHA.116.025253 PMC5262523 27986651

[174] Stoekenbroek RM Lambert G Cariou B Hovingh GK. Inhibiting PCSK9 - biology beyond LDL control. Nat Rev Endocrinol. (2018) 15:52–62. doi: 10.1038/s41574-018-0110-5 30367179

[175] Bergheanu SC Bodde MC Jukema JW. Pathophysiology and treatment of atherosclerosis: Current view and future perspective on lipoprotein modification treatment. Neth Heart J. (2017) 25:231–42. doi: 10.1007/s12471-017-0959-2 PMC5355390 28194698

[176] Pearson GJ Thanassoulis G Anderson TJ Barry AR Couture P Dayan N. Canadian cardiovascular society guidelines for the management of dyslipidemia for the prevention of cardiovascular disease in adults. Can J Cardiol. (2021) 37:1129–50. doi: 10.1016/j.cjca.2021.03.016 33781847

[177] Weaver OR Krysa JA Ye M Vena JE Eurich DT Proctor SD. Nonfasting remnant cholesterol and cardiovascular disease risk prediction in Albertans: a prospective cohort study. CMAJ Open. (2023) 11:E645–E53. doi: 10.9778/cmajo.20210318 PMC10374248 37491049

[178] Varbo A Benn M Tybjaerg-Hansen A Nordestgaard BG. Elevated remnant cholesterol causes both low-grade inflammation and ischemic heart disease, whereas elevated low-density lipoprotein cholesterol causes ischemic heart disease without inflammation. Circulation. (2013) 128:1298–309. doi: 10.1161/CIRCULATIONAHA.113.003008 23926208

[179] Nordestgaard BG Langsted A Mora S Kolovou G Baum H Bruckert E. Fasting is not routinely required for determination of a lipid profile: clinical and laboratory implications including flagging at desirable concentration cut-points-a joint consensus statement from the European Atherosclerosis Society and European Federation of Clinical Chemistry and Laboratory Medicine. Eur Heart J. (2016) 37:1944–58. doi: 10.1093/eurheartj/ehw152 PMC4929379 27122601

[180] Zilversmit DB. Atherogenesis: a postprandial phenomenon. Circulation. (1979) 60:473–85. doi: 10.1161/01.cir.60.3.473 222498

[181] Langsted A Nordestgaard BG. Nonfasting versus fasting lipid profile for cardiovascular risk prediction. Pathology. (2019) 51:131–41. doi: 10.1016/j.pathol.2018.09.062 30522787

[182] Weaver OR Ye M Vena JE Eurich DT Proctor SD. Non-fasting lipids and cardiovascular disease in those with and without diabetes in Alberta’s Tomorrow Project: A prospective cohort study. Diabetes Med. (2023) 40:e15133. doi: 10.1111/dme.15133 37171453

[183] Aguib Y Al Suwaidi J. The copenhagen city heart study (Osterbroundersogelsen). Glob Cardiol Sci Pract. (2015) 2015:33. doi: 10.5339/gcsp.2015.33 26779513 PMC4625209

[184] The Copenhagen City Heart Study. Osterbroundersogelsen. A book of ta bles with data from the first examination (1976-78) and a five year follow-up (1981-83). The Copenhagen City Heart Study Group. Scand J Soc Med Suppl. (1989) 41:1–160. 2711133

[185] Navarese EP Vine D Proctor S Grzelakowska K Berti S Kubica J. Independent causal effect of remnant cholesterol on atherosclerotic cardiovascular outcomes: A mendelian randomization study. Arterioscler Thromb Vasc Biol. (2023) 43:e373–e80. doi: 10.1161/ATVBAHA.123.319297 37439258

[186] Ji E Lee S. Antibody-based therapeutics for atherosclerosis and cardiovascular diseases. Int J Mol Sci. (2021) 22::5770. doi: 10.3390/ijms22115770 34071276 PMC8199089

[187] Di Nubila A Dilella G Simone R Barbieri SS. Vascular extracellular matrix in atherosclerosis. Int J Mol Sci. (2024) 25:12017. doi: 10.3390/ijms252212017 39596083 PMC11594217

[188] Vazquez AM Alfonso M Lanne B Karlsson KA Carr A Barroso O. Generation of a murine monoclonal antibody specific for N-glycolylneuraminic acid-containing gangliosides that also recognizes sulfated glycolipids. Hybridoma. (1995) 14:551–6. doi: 10.1089/hyb.1995.14.551 8770642

[189] Moreno E Lanne B Vazquez AM Kawashima I Tai T Fernandez LE. Delineation of the epitope recognized by an antibody specific for N-glycolylneuraminic acid-containing gangliosides. Glycobiology. (1998) 8:695–705. doi: 10.1093/glycob/8.7.695 9621110

[190] Perez A Lombardero J Mateo C Mustelier G Alfonso M Vazquez AM. Immunogenetic analysis of variable regions encoding AB1 and gamma-type AB2 antibodies from the NeuGc-containing ganglioside family. Hybridoma. (2001) 20:211–21. doi: 10.1089/027245701753179785 11604106

[191] Lopez-Requena A Mateo de Acosta C Perez A Valle A Lombardero J Sosa K. Chimeric anti-N-glycolyl-ganglioside and its anti-idiotypic MAbs: immunodominance of their variable regions. Hybrid Hybridomics. (2003) 22:235–43. doi: 10.1089/153685903322328965 14511569

[192] Lopez-Requena A De Acosta CM Moreno E Gonzalez M PuChades Y Talavera A. Gangliosides, ab1 and ab2 antibodies I. Towards a molecular dissection of an idiotype-anti-idiotype system. Mol Immunol. (2007) 44:423–33. doi: 10.1016/j.molimm.2006.02.020 16581129

[193] Lopez-Requena A Rodriguez M de Acosta CM Moreno E PuChades Y Gonzalez M. Gangliosides, Ab1 and Ab2 antibodies II. Light versus heavy chain: An idiotype-anti-idiotype case study. Mol Immunol. (2007) 44:1015–28. doi: 10.1016/j.molimm.2006.03.004 16620986

[194] Fernandez-Marrero Y Hernandez T Roque-Navarro L Talavera A Moreno E Grinan T. Switching on cytotoxicity by a single mutation at the heavy chain variable region of an anti-ganglioside antibody. Mol Immunol. (2011) 48:1059–67. doi: 10.1016/j.molimm.2011.01.008 21306777

[195] Boren J Olin K Lee I Chait A Wight TN Innerarity TL. Identification of the principal proteoglycan-binding site in LDL. A single-point mutation in apo-B100 severely affects proteoglycan interaction without affecting LDL receptor binding. J Clin Invest. (1998) 101:2658–64. doi: 10.1172/JCI2265 PMC508856 9637699

[196] Lundstam U Hurt-Camejo E Olsson G Sartipy P Camejo G Wiklund O. Proteoglycans contribution to association of Lp(a) and LDL with smooth muscle cell extracellular matrix. Arterioscler Thromb Vasc Biol. (1999) 19:1162–7. doi: 10.1161/01.ATV.19.5.1162 10323765

[197] Pillarisetti S Paka L Sasaki A Vanni-Reyes T Yin B Parthasarathy N. Endothelial cell heparanase modulation of lipoprotein lipase activity. Evidence that heparan sulfate oligosaccharide is an extracellular chaperone. J Biol Chem. (1997) 272:15753–9. doi: 10.1074/jbc.272.25.15753 9188470

[198] Wilkinson I Anderson S Fry J Julien LA Neville D Qureshi O. Fc-engineered antibodies with immune effector functions completely abolished. PloS One. (2021) 16:e0260954. doi: 10.1371/journal.pone.0260954 34932587 PMC8691596

[199] Vazquez AM Perez A Hernandez AM Macias A Alfonso M Bombino G. Syngeneic anti-idiotypic monoclonal antibodies to an anti-NeuGc-containing ganglioside monoclonal antibody. Hybridoma. (1998) 17:527–34. doi: 10.1089/hyb.1998.17.527 9890708

[200] Perez A Mier ES Vispo NS Vazquez AM Perez Rodriguez R. A monoclonal antibody against NeuGc-containing gangliosides contains a regulatory idiotope involved in the interaction with B and T cells. Mol Immunol. (2002) 39:103–12. doi: 10.1016/S0161-5890(02)00041-X 12213333

[201] Jerne NK. Towards a network theory of the immune system. Ann Immunol (Paris). (1974) 125C:373–89. 4142565

[202] Sarduy R Brito V Castillo A Soto Y Grinan T Marleau S. Dose-dependent induction of an idiotypic cascade by anti-glycosaminoglycan monoclonal antibody in apoE(-/-) mice: association with atheroprotection. Front Immunol. (2017) 8:232. doi: 10.3389/fimmu.2017.00232 28316603 PMC5334371

[203] Harding FA Stickler MM Razo J DuBridge RB. The immunogenicity of humanized and fully human antibodies: residual immunogenicity resides in the CDR regions. MAbs. (2010) 2:256–65. doi: 10.4161/mabs.2.3.11641 PMC2881252 20400861

[204] Paul S Grifoni A Peters B Sette A. Major histocompatibility complex binding, eluted ligands, and immunogenicity: benchmark testing and predictions. Front Immunol. (2019) 10:3151. doi: 10.3389/fimmu.2019.03151 32117208 PMC7012937

[205] Chiu ML Goulet DR Teplyakov A Gilliland GL. Antibody structure and function: the basis for engineering therapeutics. Antibodies (Basel). (2019) 8:55. doi: 10.3390/antib8040055 31816964 PMC6963682

[206] McCush F Wang E Yunis C Schwartz P Baltrukonis D. Anti-drug antibody magnitude and clinical relevance using signal to noise (S/N): bococizumab case study. AAPS J. (2023) 25:85. doi: 10.1208/s12248-023-00846-x 37658997

[207] Chames P Van Regenmortel M Weiss E Baty D. Therapeutic antibodies: successes, limitations and hopes for the future. Br J Pharmacol. (2009) 157:220–33. doi: 10.1111/j.1476-5381.2009.00190.x PMC2697811 19459844

[208] Delgado Roche L Acosta Medina E Fraga Perez A Becquer Viart MA Soto Lopez Y Falcon Cama V. Lipofundin-induced hyperlipidemia promotes oxidative stress and atherosclerotic lesions in New Zealand white rabbits. Int J Vasc Med. (2012) 2012:898769. doi: 10.1155/2012/898769 21977325 PMC3184413

[209] Delgado-Roche L Acosta E Soto Y Hernandez-Matos Y Olivera A Fernandez-Sanchez E. The treatment with an anti-glycosaminoglycan antibody reduces aortic oxidative stress in a rabbit model of atherosclerosis. Free Radic Res. (2013) 47:309–15. doi: 10.3109/10715762.2013.772995 23409997

[210] Delgado-Roche L Brito V Acosta E Perez A Fernandez JR Hernandez-Matos Y. Arresting progressive atherosclerosis by immunization with an anti-glycosaminoglycan monoclonal antibody in apolipoprotein E-deficient mice. Free Radic Biol Med. (2015) 89:557–66. doi: 10.1016/j.freeradbiomed.2015.08.027 26454078

[211] Brito V Mellal K Zoccal KF Soto Y Menard L Sarduy R. Atheroregressive potential of the treatment with a chimeric monoclonal antibody against sulfated glycosaminoglycans on pre-existing lesions in apolipoprotein E-deficient mice. Front Pharmacol. (2017) 8:782. doi: 10.3389/fphar.2017.00782 29163168 PMC5672559

[212] Lund J Winter G Jones PT Pound JD Tanaka T Walker MR. Human Fc gamma RI and Fc gamma RII interact with distinct but overlapping sites on human IgG. J Immunol. (1991) 147:2657–62. doi: 10.4049/jimmunol.147.8.2657 1833457

[213] Koutsogianni AD Adamidis PS Barkas F Liberopoulos E Su TC Yamashita S. Familial hypercholesterolemia and lipoprotein(a): A gordian knot in cardiovascular prevention. Metabolites. (2022) 12:1065. doi: 10.3390/metabo12111065 36355148 PMC9693181

[214] Genest J Pedersen TR. Prevention of cardiovascular ischemic events: high-risk and secondary prevention. Circulation. (2003) 107:2059–65. doi: 10.1161/01.CIR.0000067881.26274.BD 12707251

